# Supplementary material for: Catalytic trajectory of a dimeric nonribosomal peptide synthetase subunit with an inserted epimerase domain
Source: Nat Commun. 2022 Feb 1;13:592. doi: 10.1038/s41467-022-28284-x (PMC8807600; doi:10.1038/s41467-022-28284-x)
Supplement: Supplementary file 1 — Supplementary Information [file 41467_2022_28284_MOESM1_ESM.pdf]

Supplementary Information

Catalytic trajectory of a dimeric nonribosomal peptide synthetase subunit with an inserted epimerase domain

Jialiang Wang[1], Dandan Li[1], Lu Chen[1], Wei Cao[1], Liangliang Kong[2], Wei Zhang[1], Tristan Croll[3], Zixin Deng[1,*], Jingdan Liang[1,*] & Zhijun Wang[1,*]

[1]State Key Laboratory of Microbial Metabolism, Joint International Research Laboratory of Metabolic & Developmental Sciences, and School of Life Science & Biotechnology, Shanghai Jiao Tong University, Shanghai, China.

[2]National Facility for Protein Science in Shanghai, Chinese Academy of Sciences, Shanghai, 201204, China.

[3]Cambridge Institute for Medical Research, University of Cambridge, Keith Peters Building, Cambridge CB2 0XY, UK.

*Address correspondence to Zixin Deng, zxdeng@sjtu.edu.cn; Jingdan Liang, jdliang@sjtu.edu.cn or Zhijun Wang, wangzhijun@sjtu.edu.cn.

J.L.Wang and D.D.Li contributed equally to this work.

This file includes:

Supplementary Figs. 1-22

Supplementary Tables 1-3

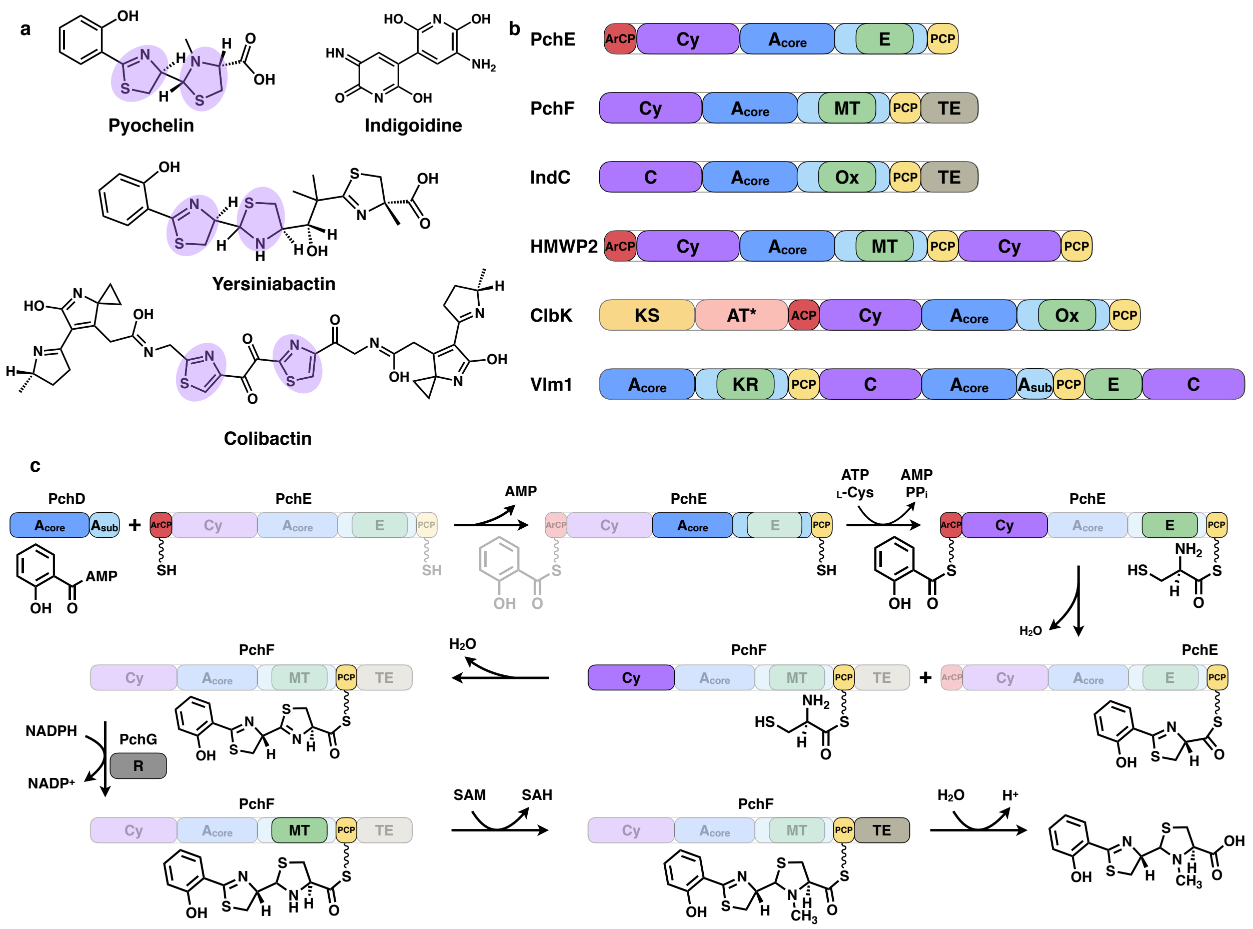

Supplementary Fig. 1 Representative NRPs, NRPSs and the biosynthetic pathway of pyochelin. a Nonribosomal peptide examples. Shared thiazoline and thiazolidine rings are highlighted with Cy purple color. **b** Domain organizations for NRPSs with tailoring domains inserted into the A domain. PchE and PchF, the two subunits that synthesize pyochelin; IndC, NRPS that forms indigoidine (blue pigment); HMWP2, high-molecular-weight protein 2 that participates in yersiniabactin (a siderophore) biosynthesis; ClbK, involved in the synthesis of colibactin (cancer-causing agent); Vlm1, valinomycin (potassium ionophore) synthetase 1. Abbreviations used for each domain: ArCP and PCP, aryl carrier protein and peptidyl carrier protein, respectively; C or Cy, condensation or cyclization domain; A, adenylation domain; TE, thioesterase; E, epimerase; MT, methyltransferase; Ox, oxidase; KR, ketoreductase. **c** The biosynthetic pathway by which PchDEFG generates pyochelin. The catalytic active domains in each reaction step are highlighted, with non-active domains shown as transparent. The reaction is initiated by PchD catalytically activating salicylate, which is subsequently transferred to the ArCP domain of PchE and condensed with an A domain-activated L-cysteine by the Cy domain. The epimerization catalyzed by the E domain is performed at this stage. The upstream intermediate is transferred to the PCP domain of PchF and condensed with the second activated L-cysteine. Mature pyochelin is released by the TE domain of PchF, after the

second thiazoline is reduced to thiazolidine by reductase (PchG). Note that the A_{sub} domain is colored lighter blue.

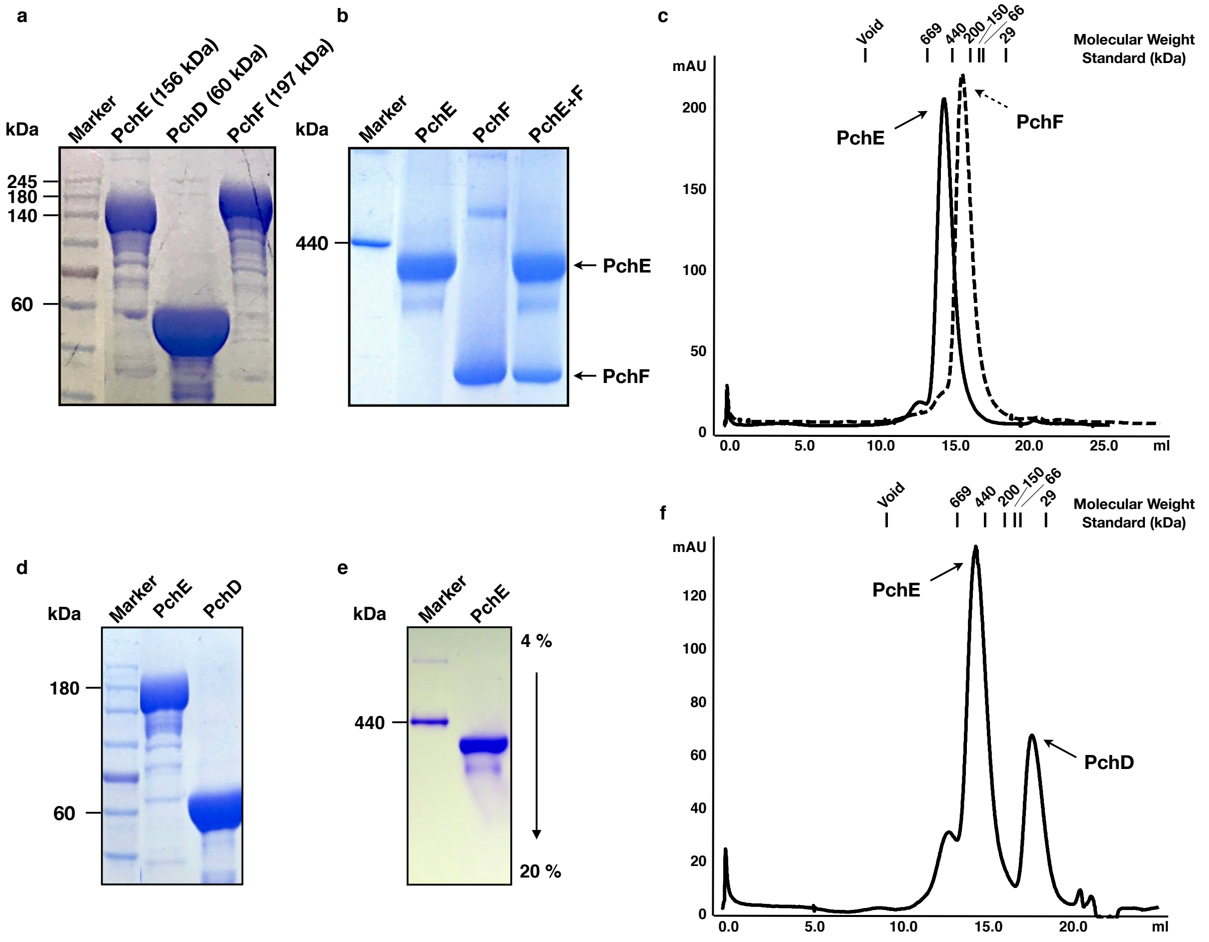

Supplementary Fig. 2 Purification and oligomeric state analysis of PchE in solution. **a** SDS-PAGE analysis of purified PchE, PchD and PchF. The predicted (theoretical) molecular weights are labeled respectively. **b** Native-PAGE analysis of PchE, PchF, and the mixture of PchE and PchF. A 4-20% gradient was used. Note that the migration of PchE in the gel is slower than that of PchF. **c** Size exclusion chromatography (SEC, Superose 6 Increase) profile of PchE and PchF. Note that PchE elutes earlier than PchF. The apparent molecular weights (WMs) of the two proteins are approximately 484 kDa (PchE) and 322 kDa (PchF). The obtained overestimated apparent WMs of PchE and PchF may be due to their elongated and dynamic conformations, because the shape and Stokes radius of the samples can vary significantly from the largely commercial globular standard proteins, which the extended shape of proteins can easily result in an anomalously earlier elution from the size exclusion chromatographic column[1,2]. Despite the overestimated apparent MWs of the two proteins, the hypothesis of PchE being homo-oligomeric is reasonable, based on the MWs comparison between Supplementary Fig. 2a and b-c. **d** SDS-PAGE analysis of purified PchE with a C-terminus Strep-tag II and PchD with an N-terminus His-tag. **e** Native-PAGE analysis of PchE. A 4-20% gradient

was used. **f** Size exclusion chromatography profile of PchE used for cryo-EM. One representative result from at least three independent experiments is shown (a, b, d, and e). Source data are provided as a Source Data file.

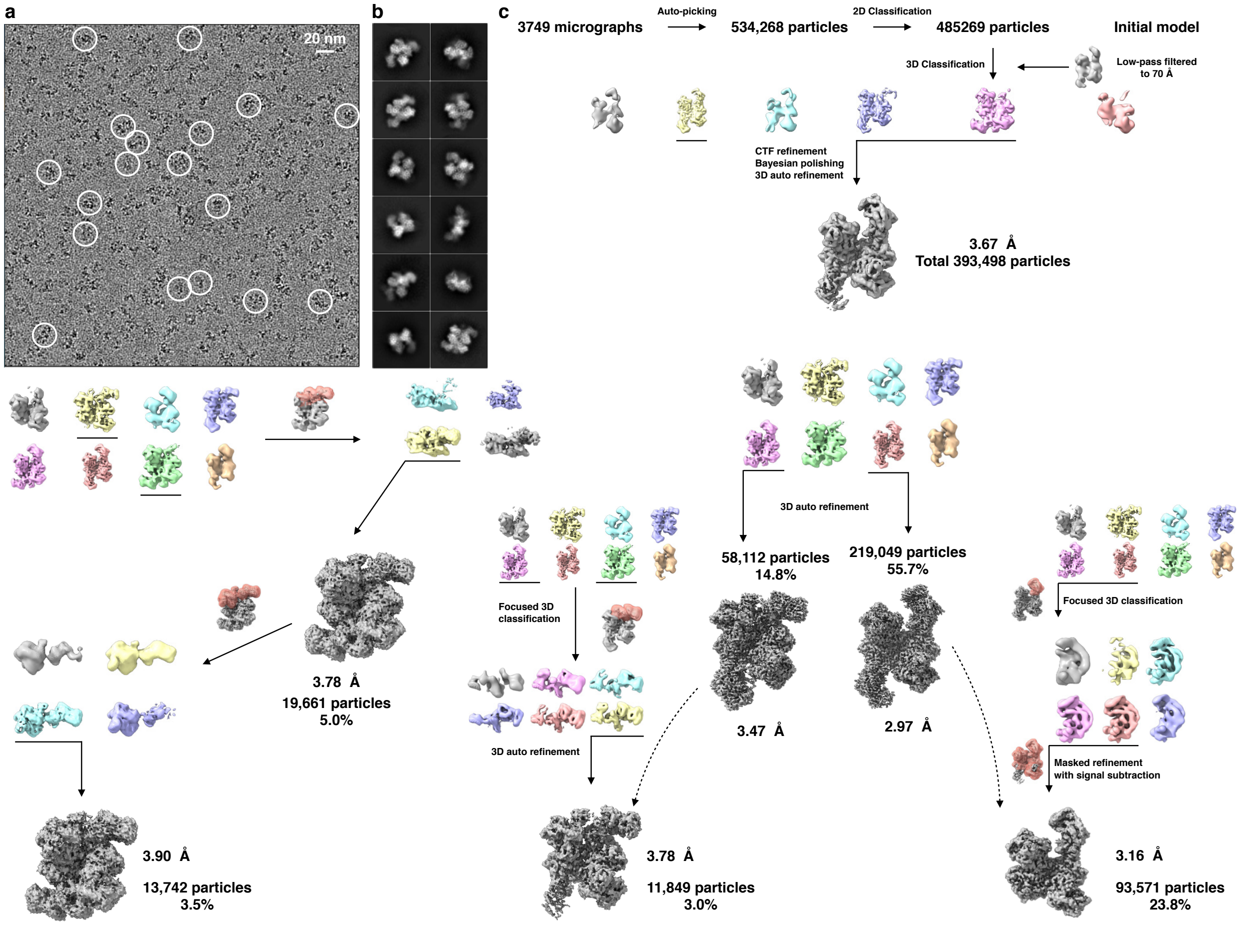

Supplementary Fig. 3 Cryo-EM structure determination of PchE. a One representative cryo-EM micrograph from the 3749 movie stacks of PchE with selected particles in white circles. Scale bar, 20 nm. At least three independent experiments were repeated with similar results. **b** Representative 2D class averages of PchE. **c** A data processing workflow for the resolution-labeled cryo-EM maps.

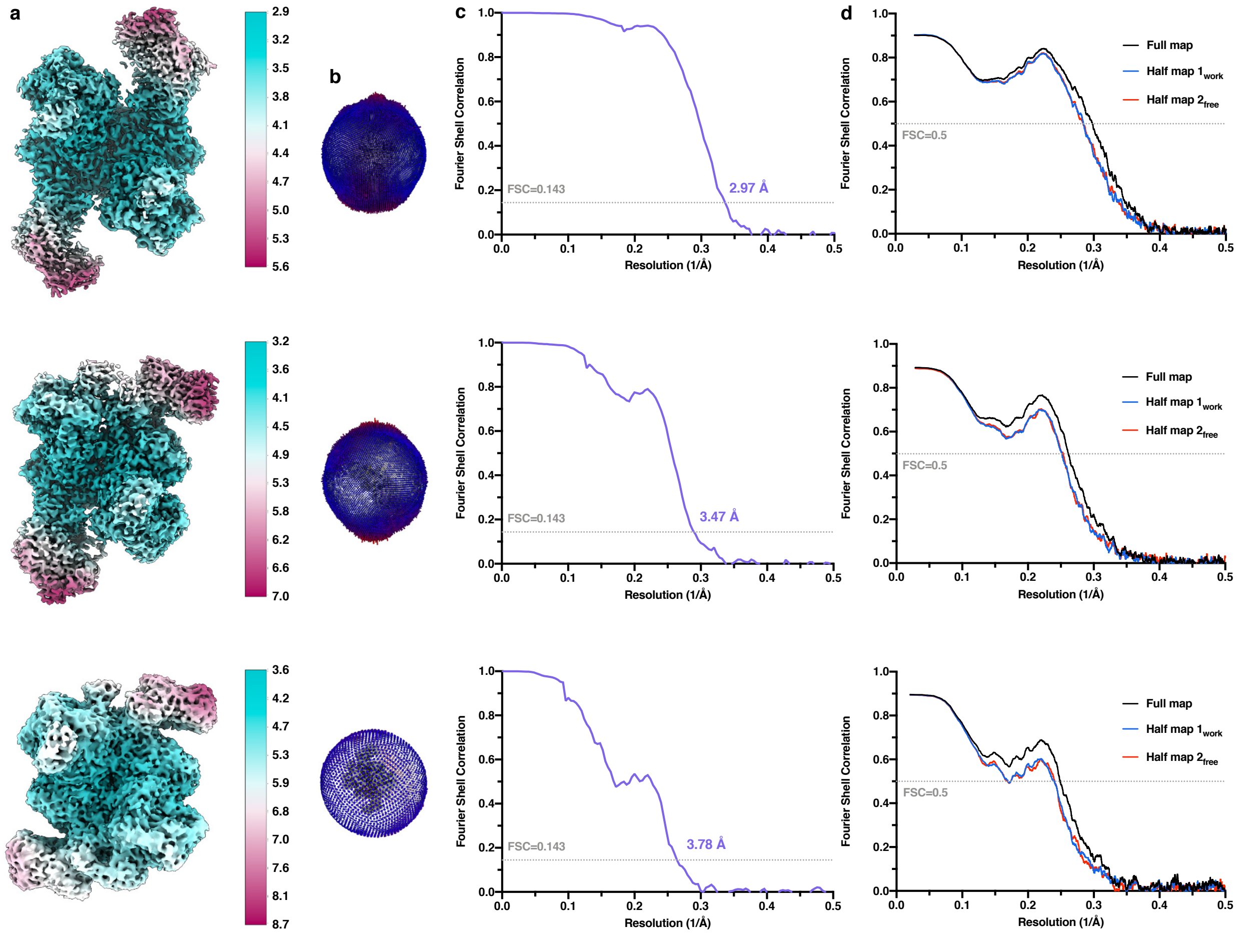

Supplementary Fig. 4 Cryo-EM analysis of PchE structures (related to Fig. 1). Top, conformation 1; middle, conformation 3; bottom, conformation 2. **a** Local resolution of each map estimated in RELION. **b** Angular distribution of all particles used for the final reconstruction of each map. **c** Gold-standard FSC curves of the resolution-labeled cryo-EM map (FSC=0.143 criterion). **d** FSC curves of the final refined model versus the map that it was refined against (black); of the model refined in the first of the two independent maps used for the gold-standard FSC versus that same map (blue); and of the model refined in the first of the two independent maps versus the second independent map (red). The small difference between the work and free FSC curves indicates that the model did not suffer from overfitting.

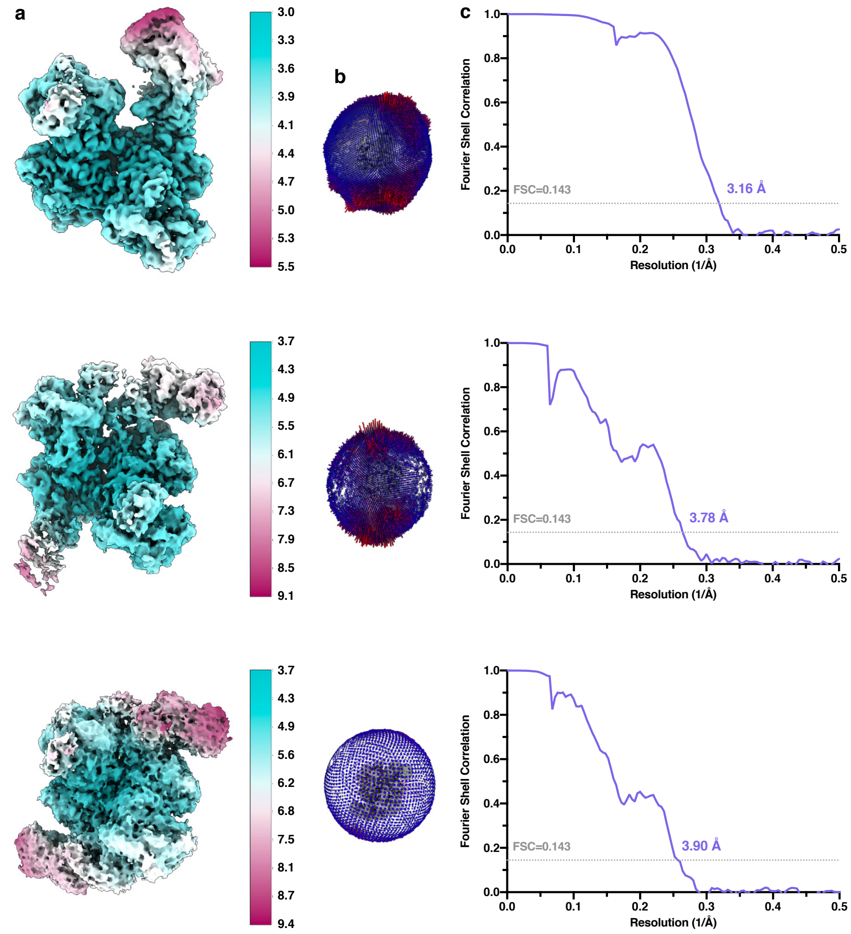

Supplementary Fig. 5 Cryo-EM analysis of PchE structures for better density integrity of moving domains (related to Supplementary Figs. 3, 4). Top, conformation 1 for the E domain; middle, conformation 3 for the PCP, rotated A_{sub} and E domains; bottom, conformation 2 for both the ArCP and PCP domains interacting with Cy. **a** Local resolution of each map estimated in RELION. **b** Angular distribution of all particles used for the final reconstruction of each map. **c** Gold-standard FSC curves of the resolution-labeled cryo-EM map (FSC=0.143 criterion).

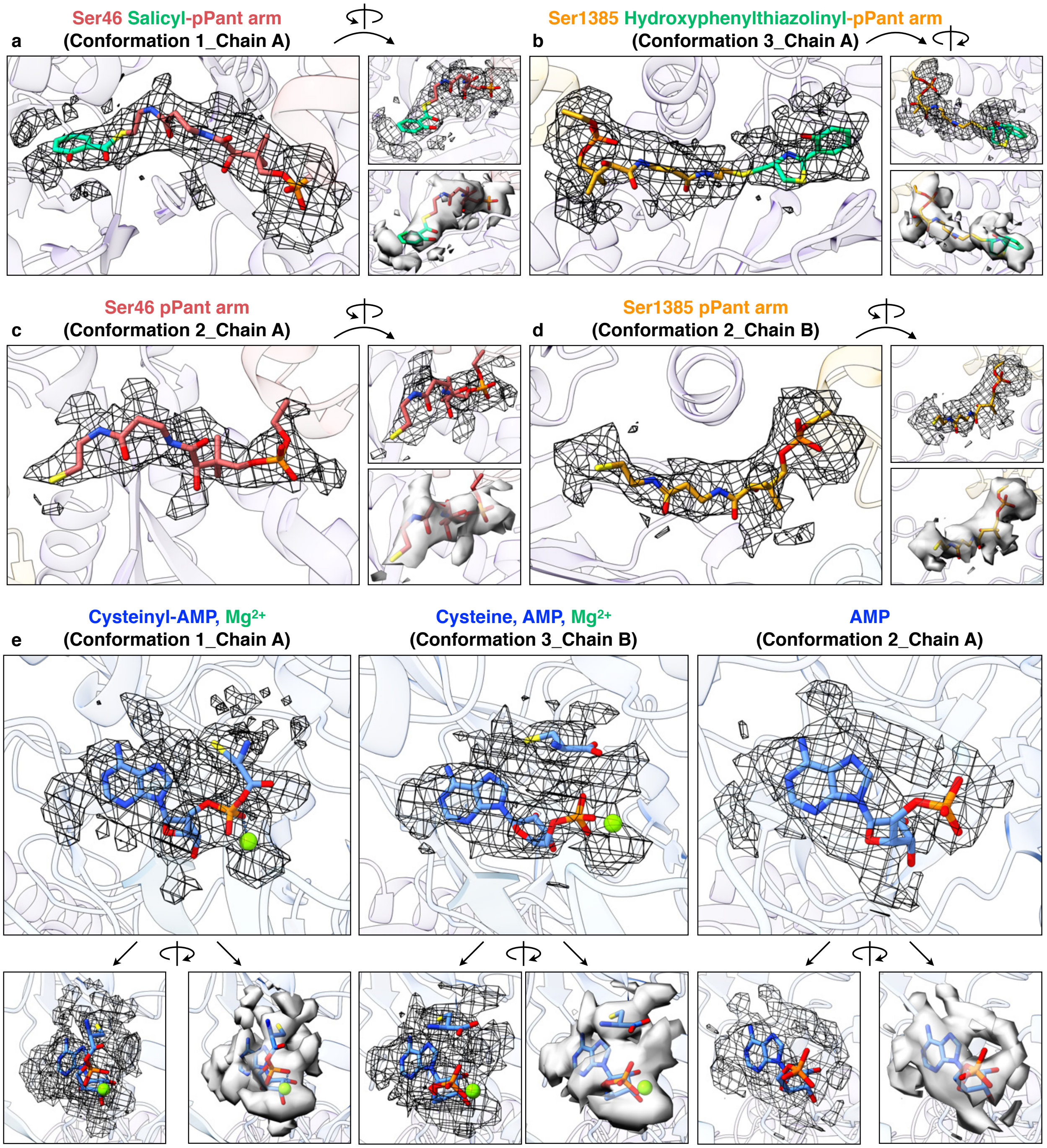

Supplementary Fig. 6 Representative cryo-EM maps of the PchE ligands. The cryo-EM maps of the ligands observed in this study, shown as meshes and surfaces in two different rotational views, are displayed at different contour levels and generated in ChimeraX. The carving distance is 3 Å. Note that the maps for the pPant intermediates are not particularly definitive for their absolute conformations. The colors and labels of distinct conformations and chains are corresponding to Fig. 1. **a** Map of the Ser46 salicyl-pPant arm of the ArCP domain in conformation 1_chain A, contoured at 0.007 (3.0σ). This ligand was also observed similarly in conformation 3_chain B. **b** Map of the Ser1385

Hydroxyphenylthiazolinyl-pPant arm of the PCP domain in conformation 3_chain A, contoured at 0.006 (2.8σ). **c** Map of the Ser46 pPant arm of the ArCP domain in conformation 2_chain A, contoured at 0.010 (4.7σ). **d** Map of the Ser1385 pPant arm of the PCP domain in conformation 2_chain B, contoured at 0.006 (2.8σ). **e** Maps were contoured at 0.012 (Left, 5.2σ; middle, 5.5σ; right, 5.7σ). Left, map of the Cysteinyl-AMP and Mg^{2+} within A domain in conformation 1_chain A (similar in chain B and conformation 3_chain A); middle, map of the Cysteine, AMP and Mg^{2+} within A domain in conformation 3_chain B; right, map of the AMP within A domain in conformation 2_chain A (similar in chain B).

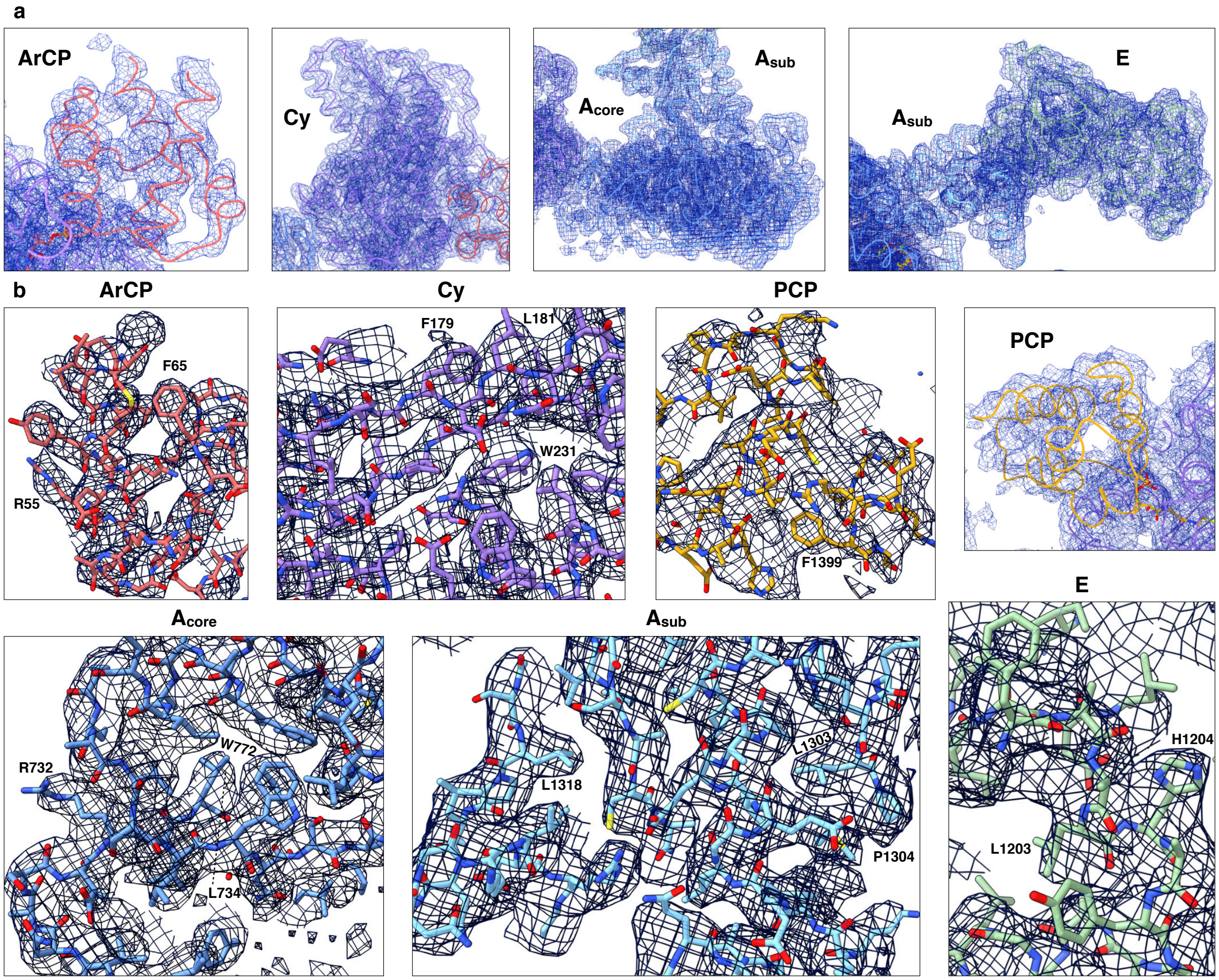

Supplementary Fig. 7 Representative cryo-EM maps of the PchE domains. The displayed map including the ArCP, Cy, A_{core}, A_{sub}, and E domains is from the conformation 1_chain A structure. The map of the PCP domain is from conformation 3_chain A structure. The maps are shown as meshes, contoured at 0.012 (5.2σ) for the Cy and A_{core} domains, and 0.010 (4.3σ) for the ArCP, A_{sub}, and E domains, and 0.010 (4.4σ) for the PCP domain. The figure was generated in ChimeraX. **a** The cryo-EM maps for each of the PchE domains, fitted with the cartoon represented atomic models (shown as lines). **b**

Close-up views of the regions of each domain, fitted with the full-atom represented atomic models (shown as sticks).

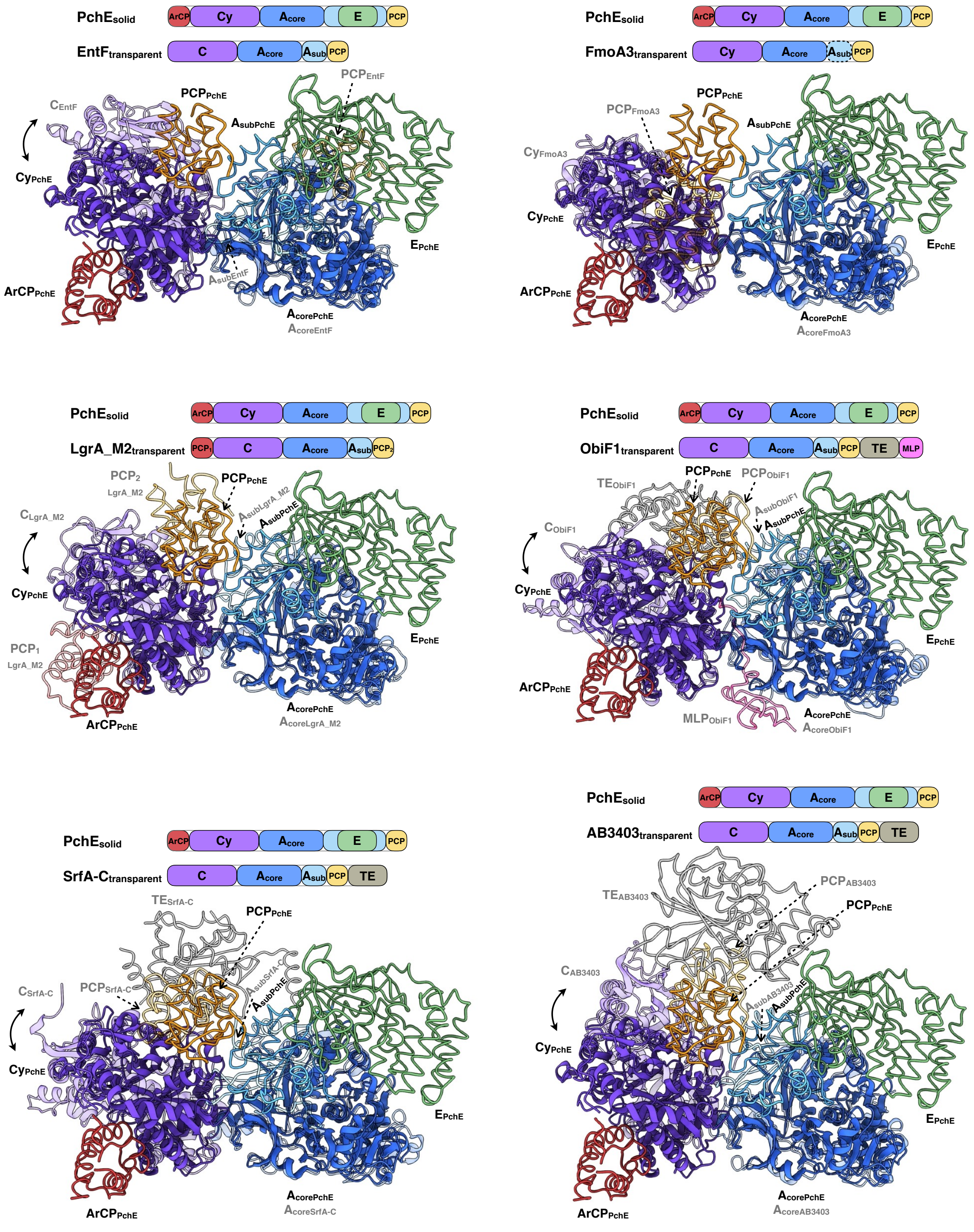

Supplementary Fig. 8 Comparisons of the Cy:A_{core} (C:A_{core}) conformations and domain positions. Superposition of the PchE structure (conformation 3_chain A) with

FmoA3[3] (PDB 6LTA), EntF[4] (PDB 5T3D), LgrA_M2[5] (PDB 6MFZ), ObiF1[6] (PDB 6N8E), SrfA-C[7] (PDB 2VSQ), and AB3403[4] (PDB 4ZXH) on the A_{core} domain. The linear organization for each comparison is shown above, with all the domains labeled. The relative stable Cy:A_{core} (C:A_{core}) domains are displayed as ribbons, and the other dynamic domains are shown as lines. For each superposition, the PchE structure is colored solid with the comparison structure shown as transparent. The double-headed arrows indicate the relative rotation between the position of PchE Cy domain and C domain of five NRPS modules.

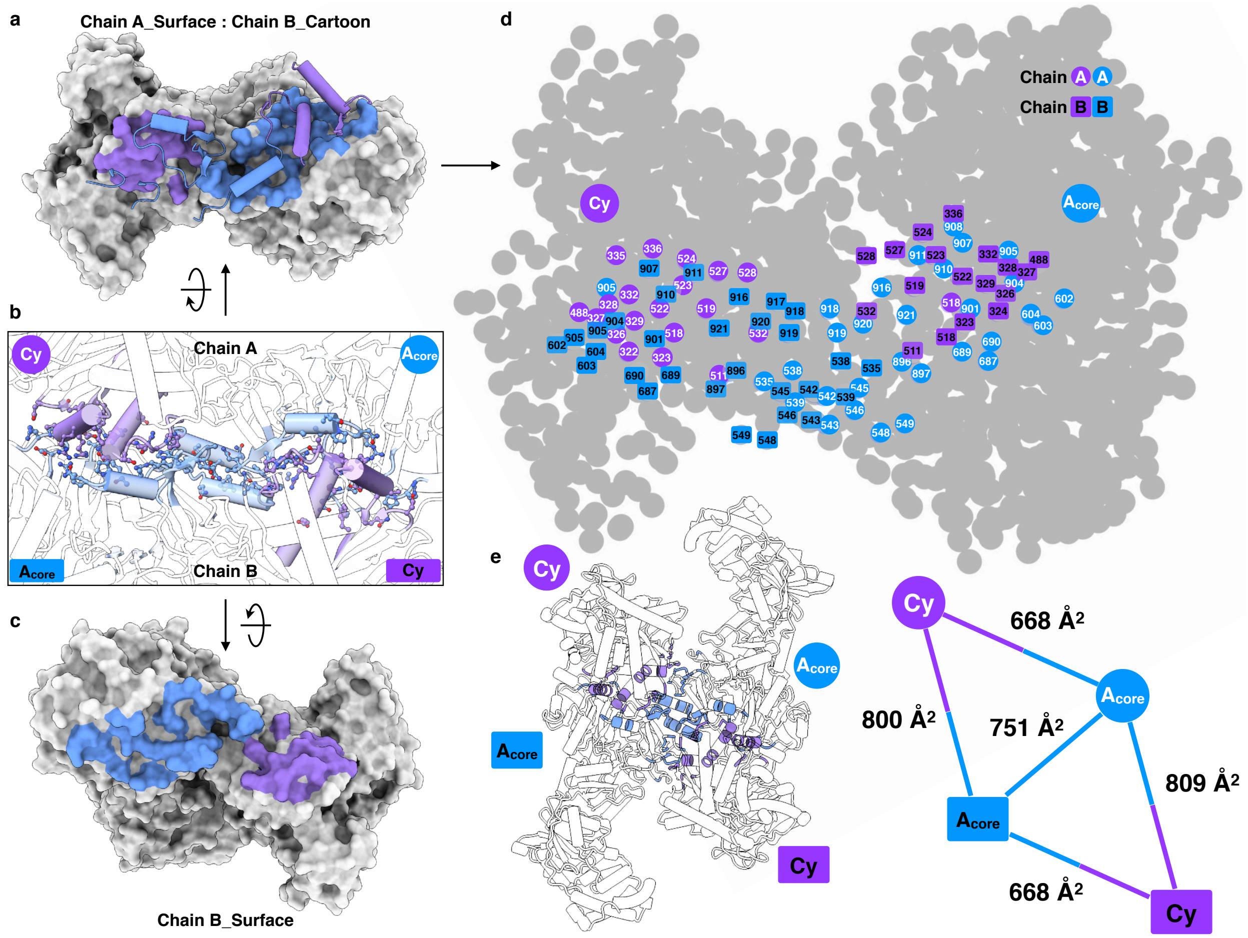

Supplementary Fig. 9 Interface of dimeric PchE. a Schematic showing contacts between chains in which chain A is shown as surface and chain B as ribbon cartoon representations. The contacting region is emphasized with color. **b** Close-up view of the PchE dimer interface reveals the Cy-A_{core} antiparallel "head-to-tail" contact pattern. The interacting residues are shown as sticks and highlighted with colors. **c** Schematic showing contacts between chains with only chain B shown as a surface representation. **d** Detailed view of the contact residues labeled with black and white residue numbers for chains A and B, respectively. **e** Surface buried between and within the two monomer chains.

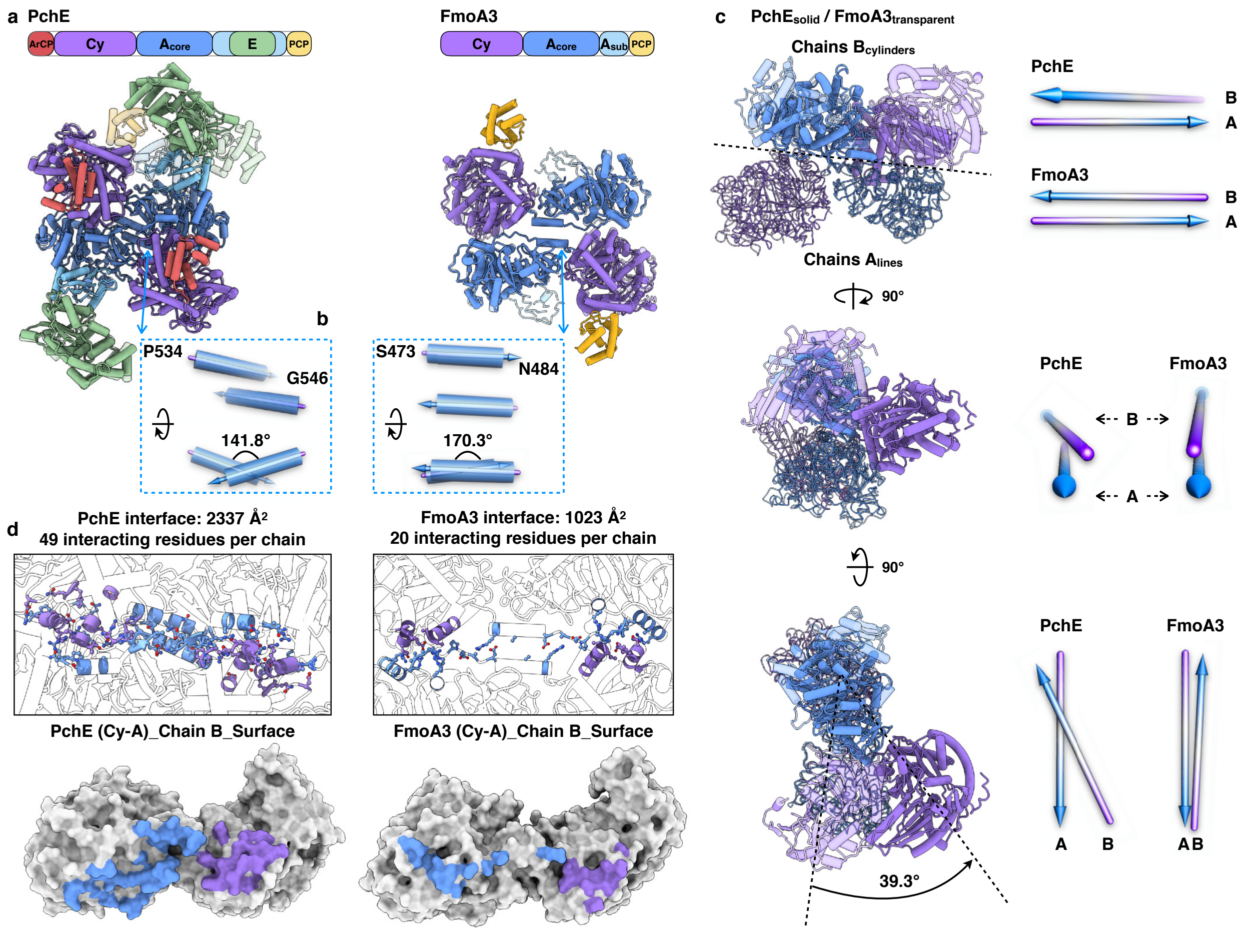

Supplementary Fig. 10 Structural comparisons of the dimeric organization between PchE and FmoA3[3]. a Comparison of the two overall architectures and linear organizations. **b** Comparison of the two inter-helix arrangements. Close-up views of interface helices (with angles labeled) are bordered with dashed lines. **c** Comparison of the two $(Cy\text{-}A_{core})_{chain\ A}\text{-}(A_{core}\text{-}Cy)_{chain\ B}$ quadrangular stable cores, shown in front, side, and top views (top panel to bottom, respectively). Left, chains A of PchE and FmoA3 are superposed and shown as lines, with chains B shown as cylinders for comparison purpose. The tilt angle between chain B of PchE and FmoA3 is labeled (bottom). Right, schematics showing the inter-chain arrangements of PchE and FmoA3. **d** Comparison of the two dimeric interfaces. The interacting residues are colored and shown as sticks. Bottom, Schematics showing contacts between chains with only chains B shown as surface representation.

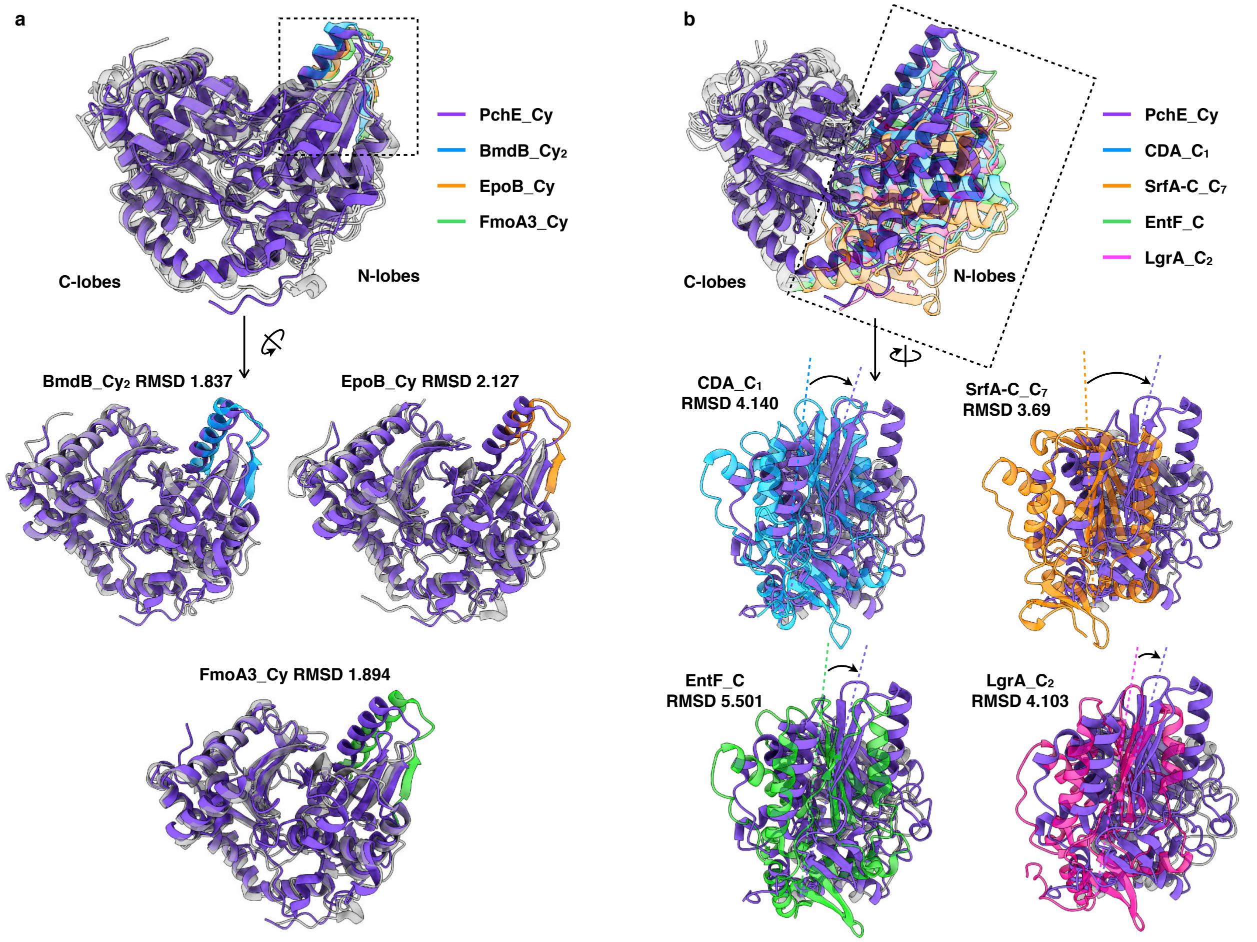

Supplementary Fig. 11 Structural comparisons of Cy and C domain conformations. Top, superposition of PchE Cy domain with other NRPSs Cy domains (**a**) and C domains (**b**) on the C-lobes. Bottom, individual superposition of PchE Cy domain with BmdB[8] (PDB 5T3E), EpoB[9] (PDB 5T81), and FmoA3[3] (PDB 6LTA) Cy domains (**a**); and with CDA[10] (PDB 4JN3), SrfAC[7] (PDB 2VSQ), EntF[4] (PDB 5T3D), LgrA[5] (PDB 6MFW) C domains (**b**). PchE Cy domain is colored purple, other Cy and C domains are shown in gray (transparent), with regions variously colored and bordered for highlighting the structural differences (loop linked β-sheet and α-helix for the Cy domains, and the entire N-lobes for the C domains). The relative rotations between the N-lobe of C domains with PchE Cy domain are highlighted, showing the inter-lobe conformational variability.

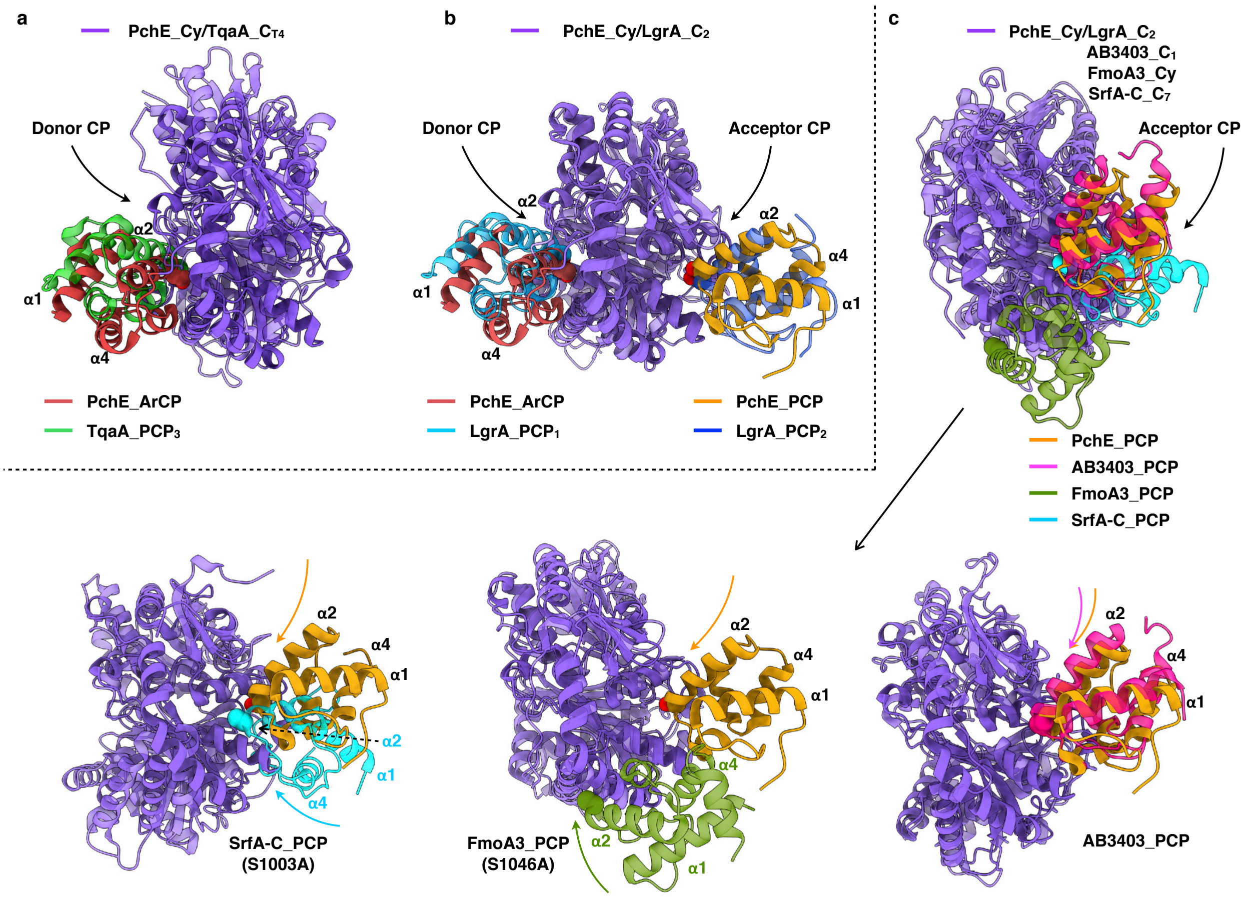

Supplementary Fig. 12 Structural comparisons of CP:Cy/C binding. All structures are superposed onto the PchE Cy domain for CP domains binding comparison at the donor site (**a**), acceptor site (**c**) and both sites (**b**). PchE ArCP:Cy:PCP domains are colored red, purple and yellow, respectively. Other NRPSs CPs are variously colored (transparent) for highlighting the CPs binding differences between PchE and TqaA[11] (**a**, PDB 5EJD); LgrA[5] (**b**, PDB 6MFZ); and AB3403[4] (**c**, PDB 4ZXH), FmoA3[3] (**c**, PDB 6LTA), and SrfA-C[7] (**c**, PDB 2VSQ). The α1, 2, and 4 helices of CP domains are labeled and the pPant-attached Ser residues (or Ser-Ala) are shown as balls.

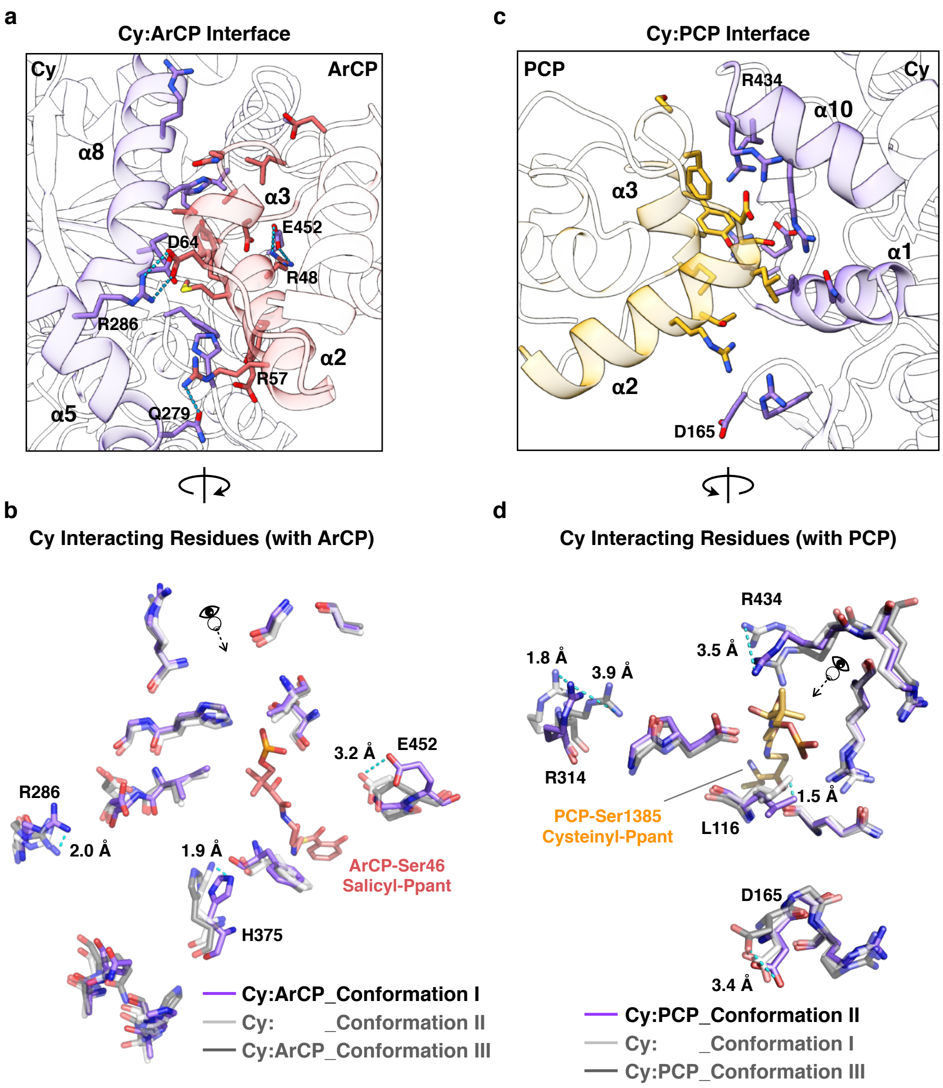

Supplementary Fig. 13 Interaction of Cy with ArCP or PCP domains reveals the small-scale dynamics of the interface residues. The ArCP-Cy (**a**) and PCP-Cy (**c**) domain interactions. The interacting secondary structures and residues are labeled and highlighted by color. The residues of the Cy domain in three conformations that interact with ArCP (**b**) or PCP (**d**) are superimposed. The subtle motion of residues is indicated with labeled distances and highlighted with dashed lines. Ser46-salicyl-pPant and Ser1385-cysteinyl-pPant are shown as sticks that indicate the entry regions of ArCP or PCP domains, respectively.

a

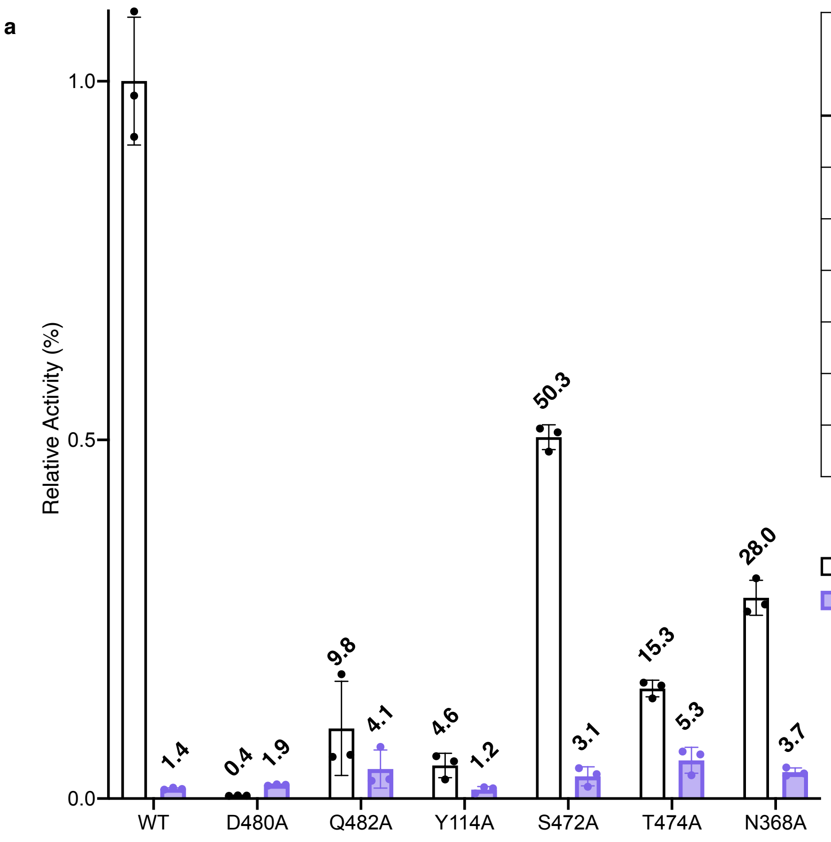

	The average peak area of linear to heterocyclic product ratio (-fold per mutant)
WT	0.014
D480A	4.634
Q482A	0.424
Y114A	0.271
S472A	0.062
T474A	0.349
N368A	0.132

b

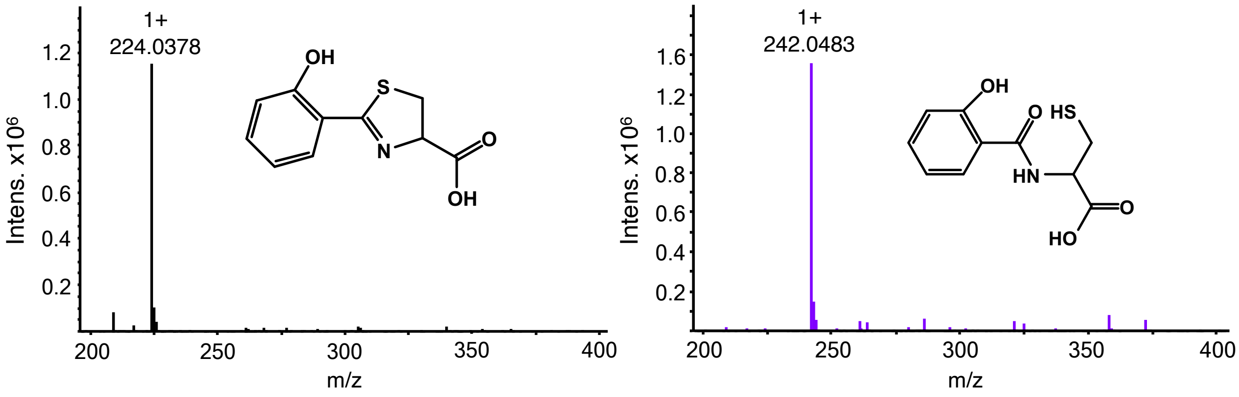

Supplementary Fig. 14 Activity assay of structure-guided mutations in the Cy domain. a The plot shows relative activities of the heterocyclic (black) and linear (purple, the intermediate) products of the key residue mutants in the Cy domain (as % of the average peak area of WT heterocyclic product). The product ratios of linear to heterocyclic are labeled in the right table (as -fold of the average peak area per mutant). Data are presented as mean values ± SD from three biologically independent experiments (*n*=3). Source data are provided as a Source Data file. **b** Representative mass spectra of the heterocyclic (left, black peak) and linear product (right, purple peak) in positive ion mode. The heterocyclic product has calculated and experimentally

determined m/z $[M+H]^+$ values of 224.0376 and 224.0378, and the linear product has calculated and experimentally determined m/z $[M+H]^+$ values of 242.0482 and 242.0483.

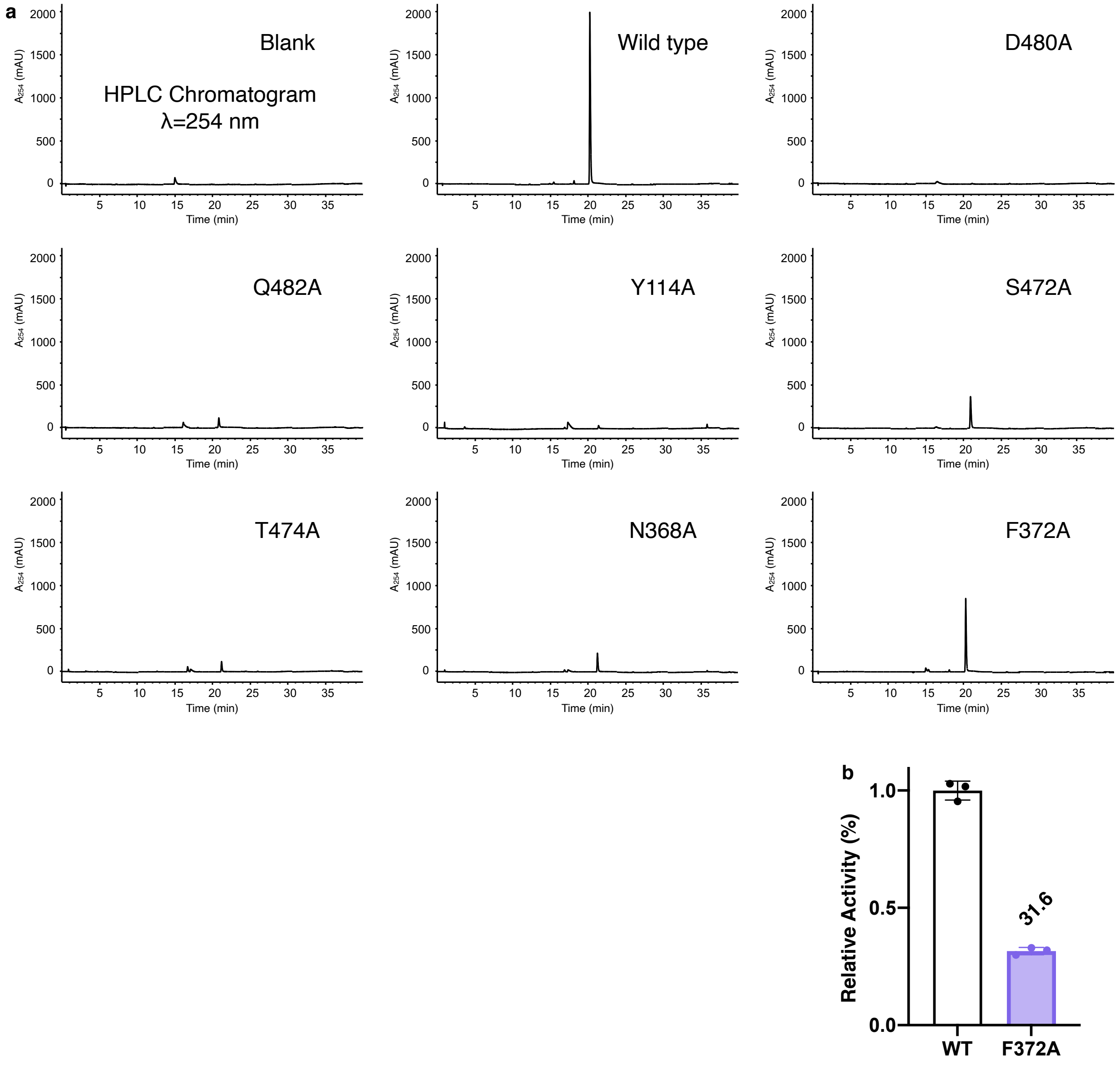

Supplementary Fig. 15 Representative HPCL chromatogram profiles of all the PchE mutants. a Representative HPLC traces at *λ*=254 nm showing the products of the *in vitro* reactions catalyzed by PchD with PchE or PchE mutants. SrfD, an external thioesterase enzyme involved in surfactin biosynthesis by *Bacillus subtilis*[12], was included in the reaction mixture to release the products. **b** The plot shows relative activities of F372A in the PchE Cy domain. Data are presented as mean values ± SD from three biologically independent experiments (*n*=3). Source data are provided as a Source Data file.

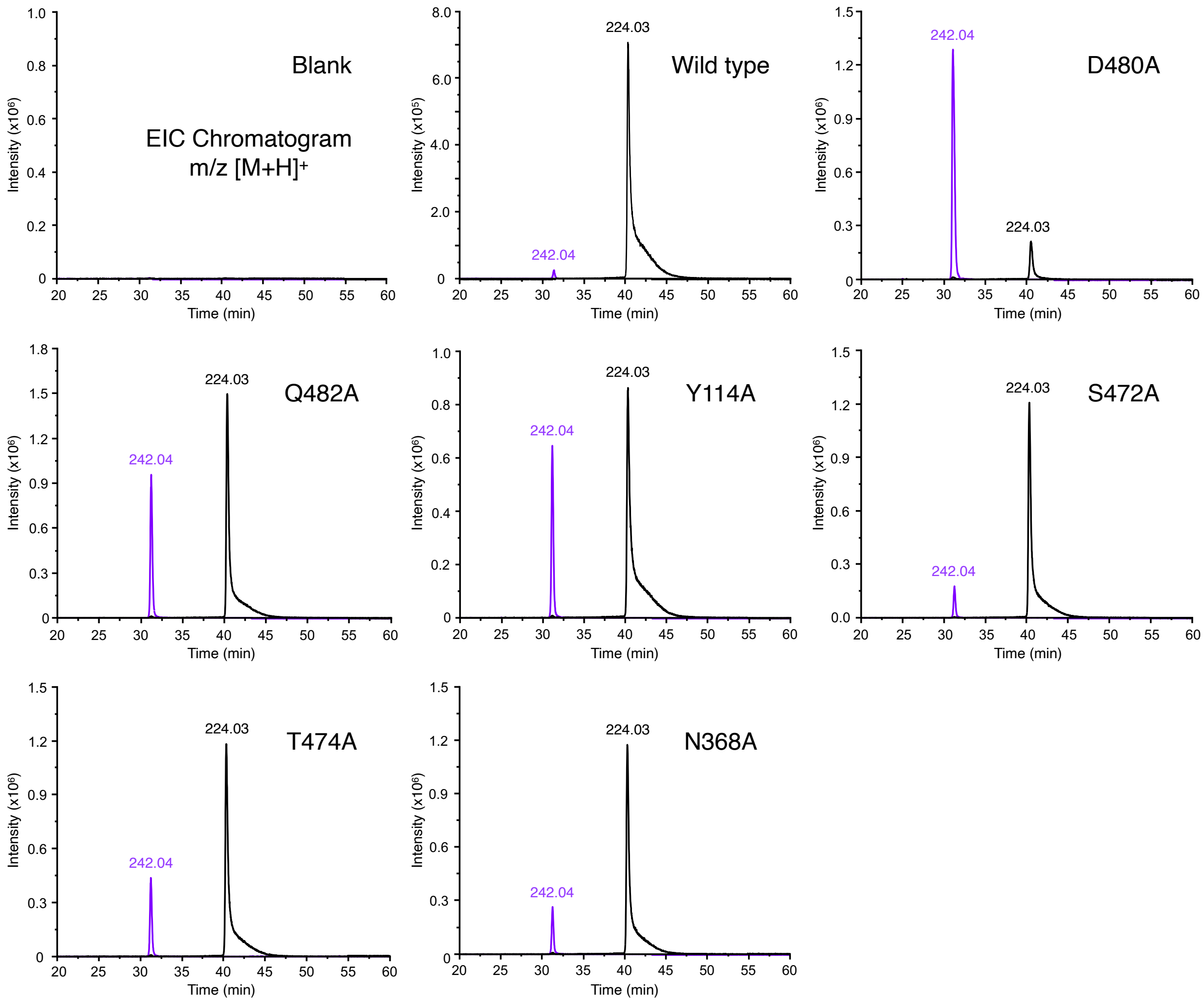

Supplementary Fig. 16 Representative extracted ion chromatogram profiles of the key mutants in the Cy domain. Representative EIC traces in positive ion mode showing the heterocyclic (black) and linear (purple) products of the *in vitro* reactions catalyzed by PchD with PchE or PchE mutants, which the products were released by the thioesterase SrfD. The selective m/z $[M+H]^+$ values used to detect the products are labeled over the traces, and the heterocyclic and linear products have the calculated m/z $[M+H]^+$ values of 224.03 and 242.04, respectively.

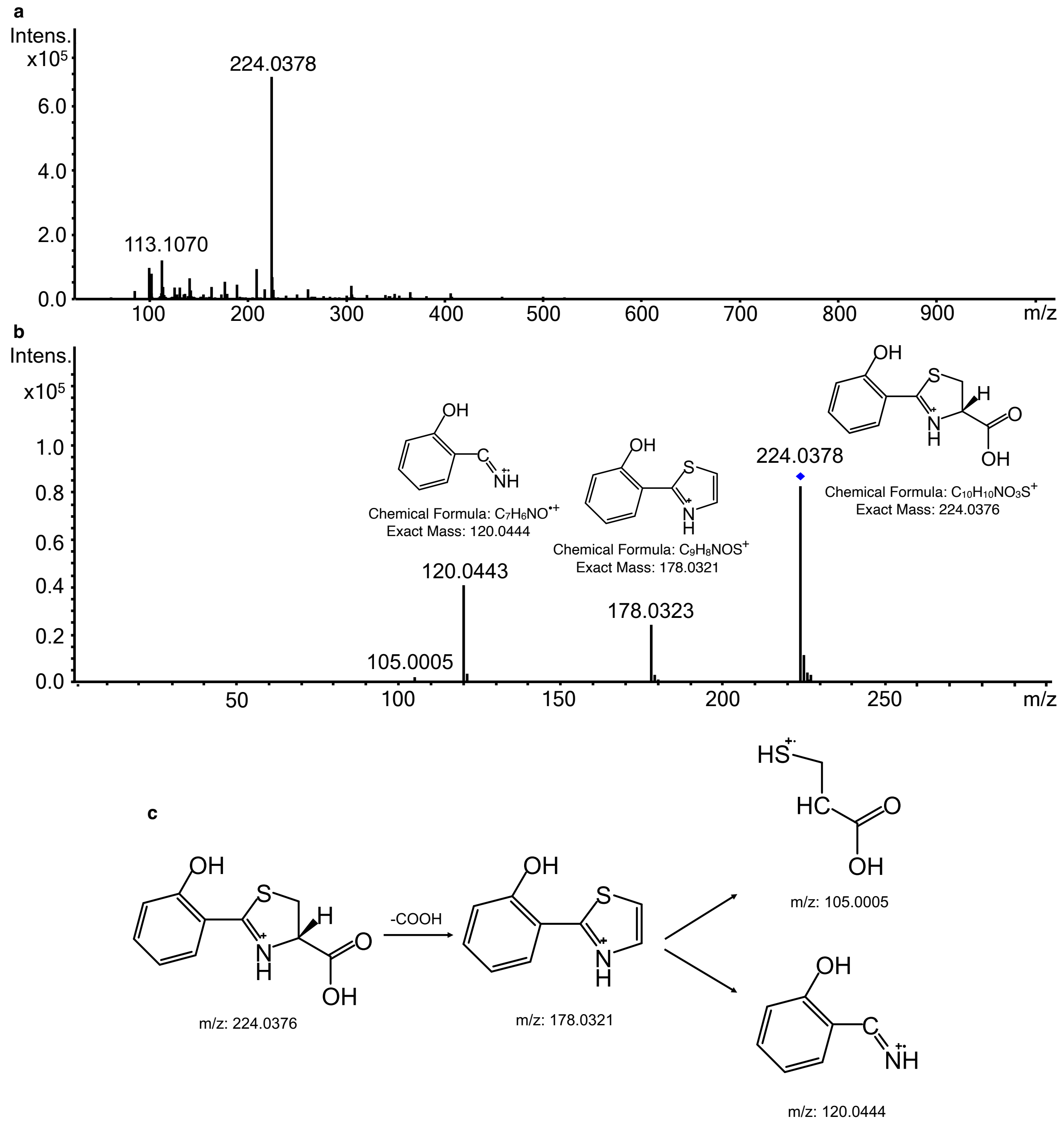

Supplementary Fig. 17 Representative mass spectrum profiles. a Representative mass spectrum of the heterocyclic product (HPT-COOH) in positive ion mode. HPT-COOH has calculated and experimentally determined m/z $[M+H]^+$ values of 224.0376 and 224.0378. **b** Representative tandem mass spectrum (MS/MS) fragmentation of HPT-COOH, with the putative fragmentation pathway (**c**).

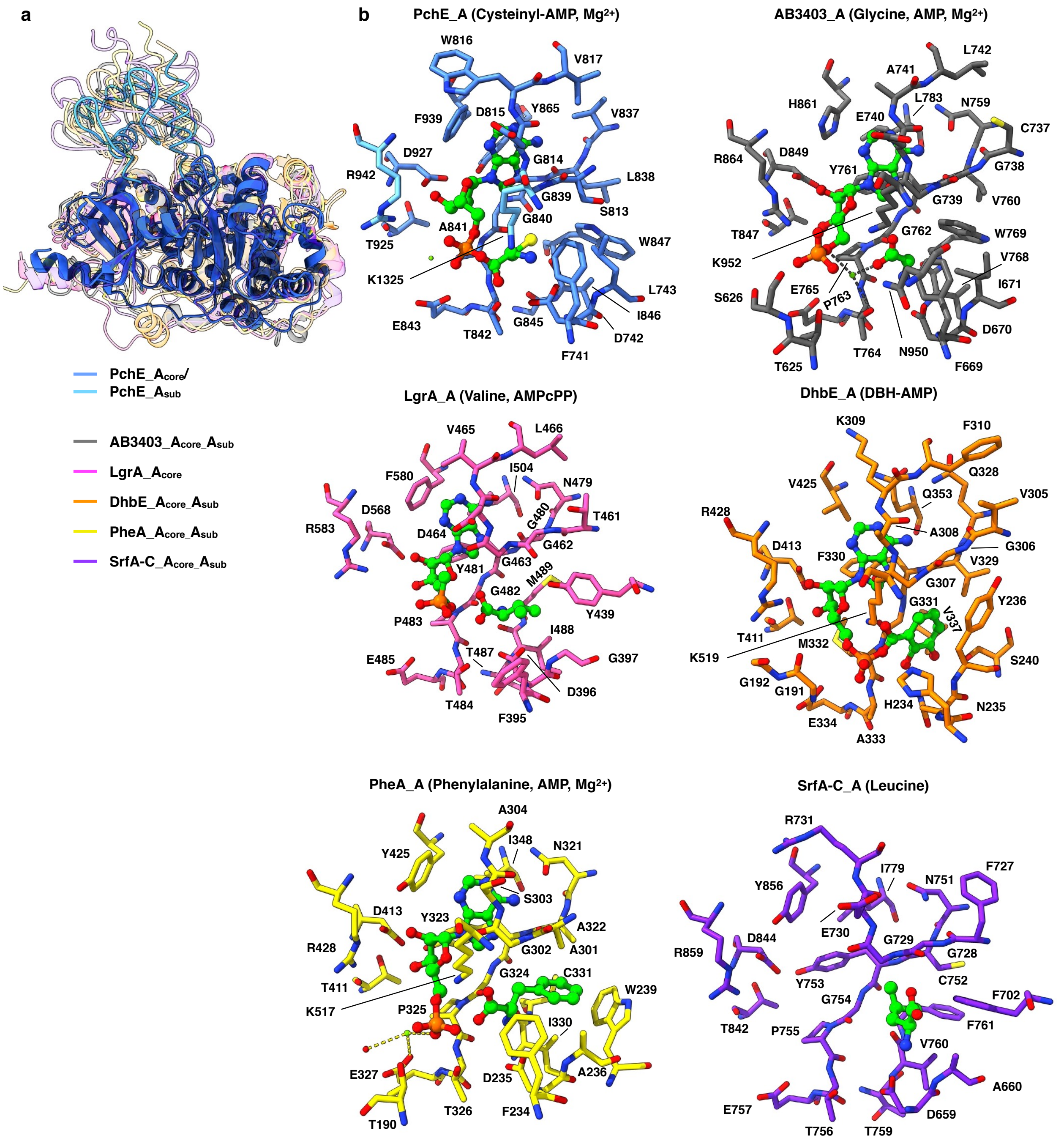

Supplementary Fig. 18 Structural comparison of A domains. a Superposition of PchE A domain with other NRPSs A domains on the A_{core} (ribbons, transparent), with the A_{sub} domain shown as lines. **b** The active sites of PchE, AB3403[4] (PDB 4ZXH), LgrA[13] (PDB 5ES7), DhbE[14] (PDB 1MDB), PheA[15] (PDB 1AMU), and SrfA-C[7] (PDB 2VSQ) A domains, shown as sticks, with the ligands colored green.

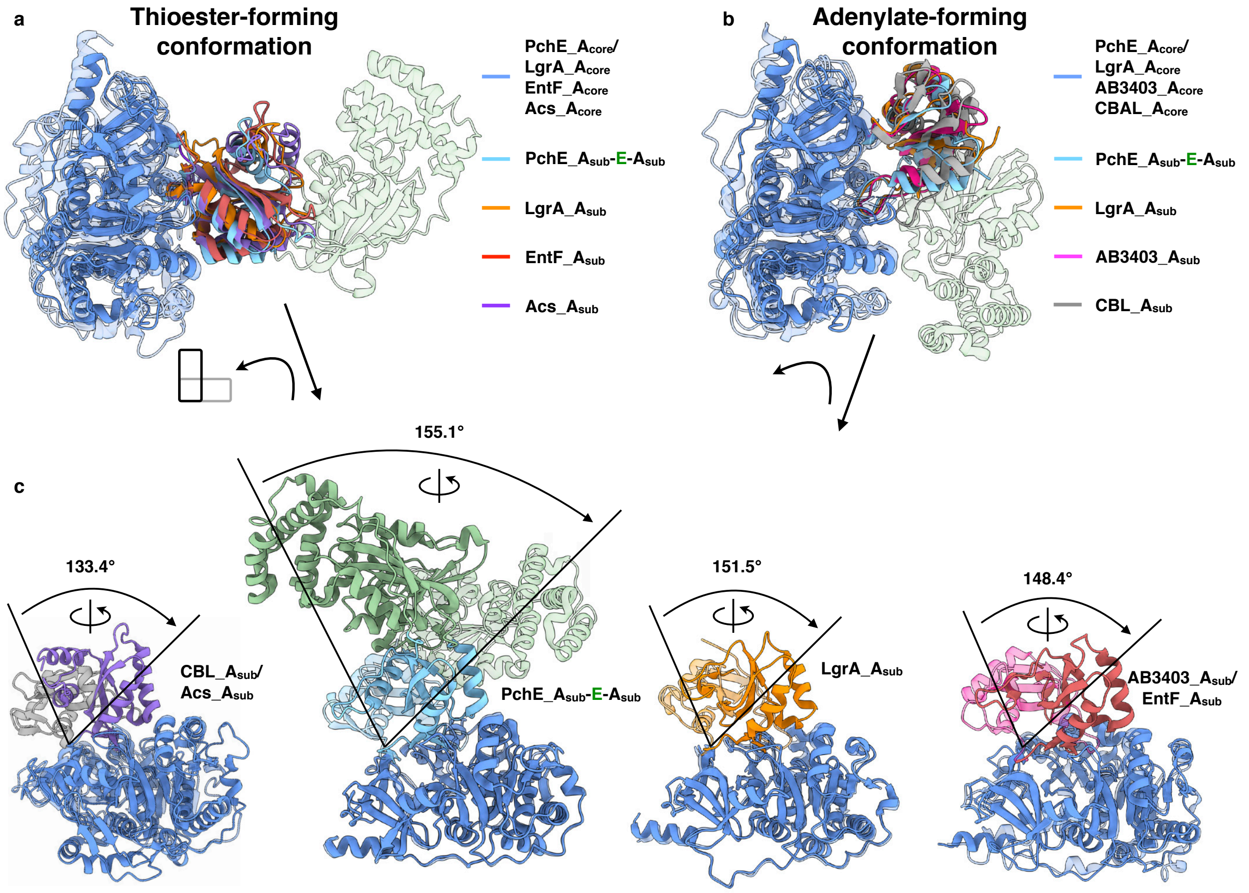

Supplementary Fig. 19 Structural comparisons of the small C-terminal A_{sub} domain rotation. All structures are superposed onto the PchE A_{core} domain for A_{sub} domains rotation comparison. The A_{core} domains are colored blue with structures of comparison shown as transparent, the A_{sub} domains are highlighted in distinct colors, and the embedded E domain of PchE are colored green (transparent). **a** Superposition of the thioester-forming conformations of PchE A domain structure with LgrA[13] (PDB 5ES8), EntF[4] (PDB 5T3D), and Acs[16] (Acetyl-CoA synthetase, PDB 1PG4) structures. **b** Superposition of the adenylate-forming conformations of PchE A domain structure with LgrA[13] (PDB 5ES5), AB3403[4] (PDB 4ZXH), and CBL[17] (4-Chlorobenzoate:CoA ligase, PDB 1T5H) structures. **c** Individual superposition of different adenylating enzyme structures reveals the similar C-terminal subdomain movements. The rotation angles were reported by DynDom software[18], and the direction of the arrowed lines indicate the catalytic states transitions from adenylate-forming to thioester-forming.

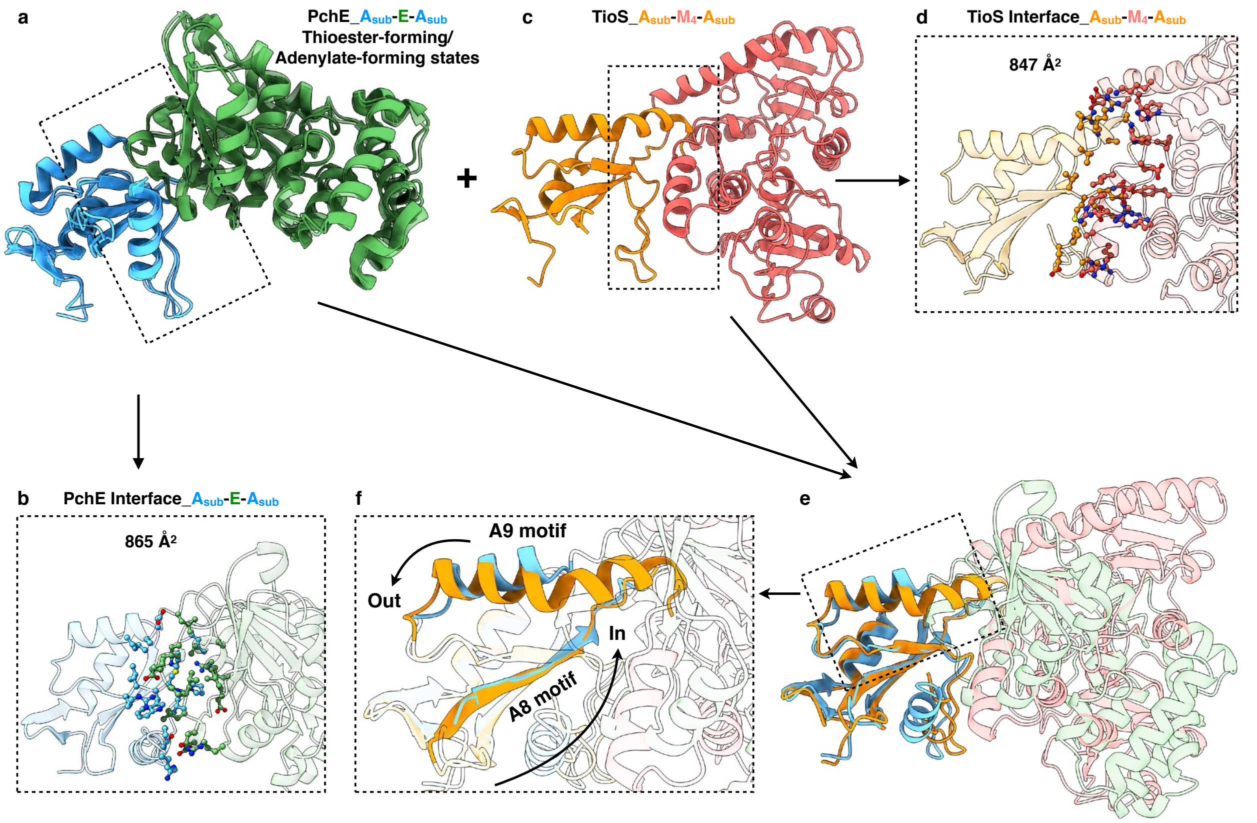

Supplementary Fig. 20 Structural comparisons of the tailoring domain-embedded didomains of PchE and TioS[19]. a Superposition of the A_{sub}-E-A_{sub} didomains of the two conformations of PchE. The small difference between them indicates that they rotate as a rigid body. **b** The interface between A_{sub} and E domains of PchE, with the contacting residues shown as balls. **c** Cartoon representation of A_{sub}-M-A_{sub} didomain of TioS[19] (PDB 5WMM) structure, with the interface residues (**d**) shown as balls. **e** Superposition of the tailoring domain-embedded structures on the interrupted A_{sub} domains, with the close-up views of the insertion sites (**f**).

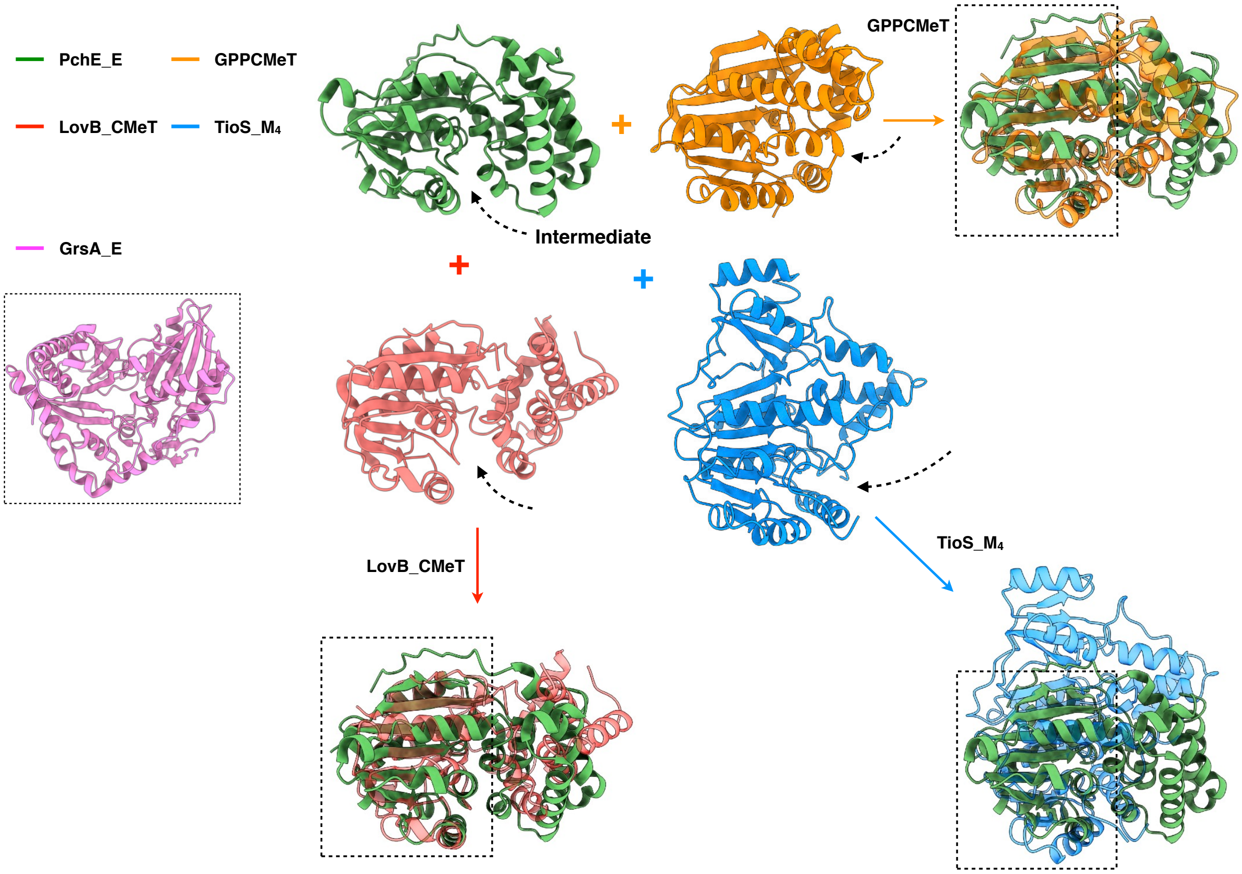

Supplementary Fig. 21 Structural comparisons of the PchE noncanonical E domain, the C superfamily canonical E domain, and the methyltransferase (MT) domains. Superposition of PchE E domain (green) with MT domains on the SAM-binding regions (dashed line bordered) of GPPCMeT[20] (geranyl diphosphate *C*-methyltransferase, PDB 3VC2), LovB_CMeT[21] (lovastatin nonaketide synthase *C*-methyltransferase, PDB 7CPX), and TioS_M_4[19] (PDB 5WMM) domains. The substrate entrances are indicated by arrows. The canonical epimerase of GrsA[22] (PDB 5ISX) structure is dashed line bordered and colored pink, showing the structural difference between the C domain superfamily epimerase and noncanonical epimerase of PchE.

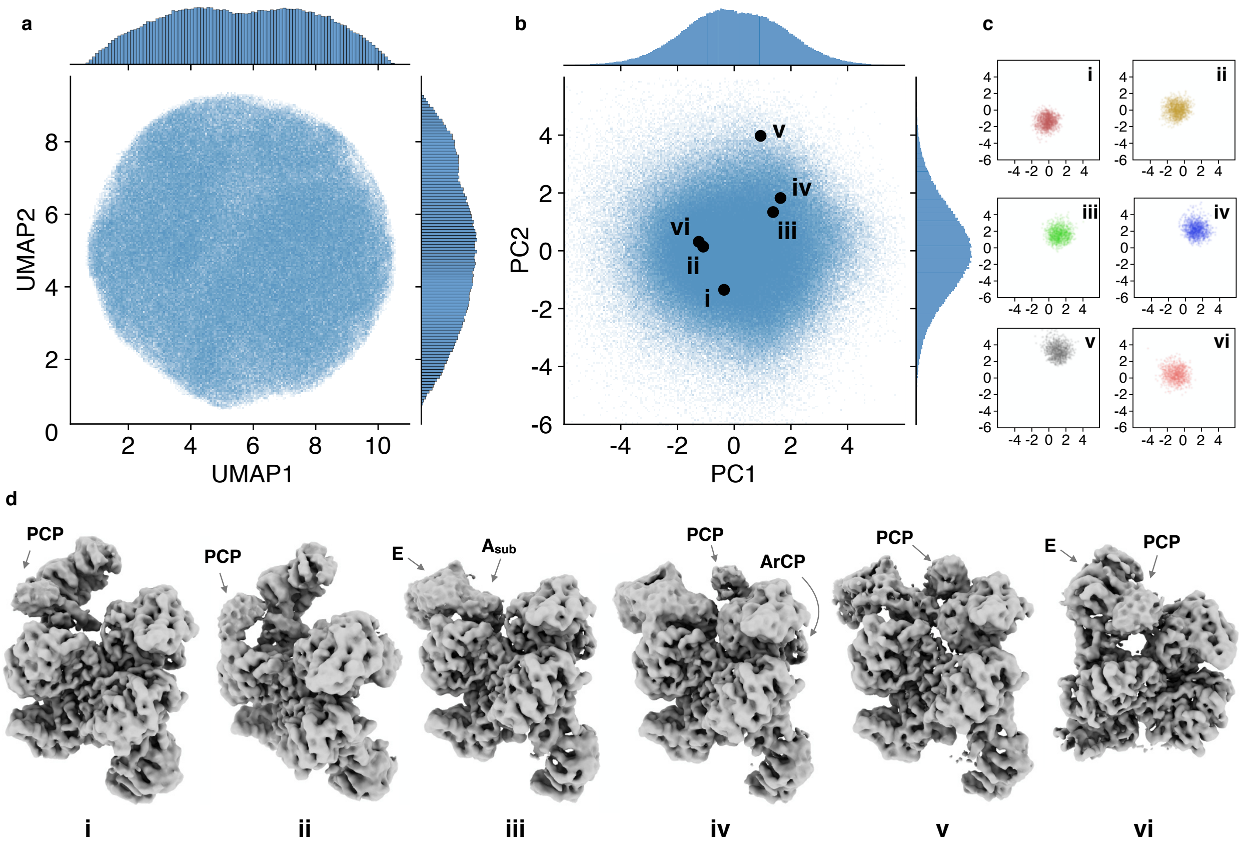

Supplementary Fig. 22 Structural heterogeneity in the PchE dataset analyzed by using cryoDRGN software (related to Fig. 5 and Supplementary Movie 2). a UMAP visualization of the latent space representation of particle images of PchE after training an 8-D latent variable model with cryoDRGN. **b** PCA projection of the 8-D latent encodings from cryoDRGN with 6 representative sample points. **c** Subplots of the localized accumulations of six representative cryoDRGN reconstructions. **d** Cryo-EM maps generated at points shown in (**b**). These maps are docked with atomic models of each domain in Fig. 5.

Supplementary Table 1. Cryo-EM data collection, processing and validation statistics.

	Conformation 1 (Substrate donation)		Conformation 2 (Condensation)		Conformation 3 (Post-Condensation)	
	EMD-31198 PDB 7EMY	EMD-31201	EMD-31200 PDB 7EN2	EMD-31203	EMD-31199 PDB 7EN1	EMD-31202
**Data collection and processing**						
Magnification	22500					
Voltage (kV)	300					
Electron exposure (e-/Å²)	60.8					
Defocus range (μm)	-1.5 to -2.5					
Pixel size (Å)	1.00					
Symmetry imposed	C1					
Final particle images (no.)	219,049	93,571	19,661	13,742	58,112	11,849
Map resolution (Å)	2.97	3.16	3.78	3.90	3.47	3.78
FSC threshold	0.143					
Map resolution range (Å)	2.9-5.6	3.0-5.5	3.6-8.7	3.7-9.4	3.2-7.0	3.7-9.1
Map sharpening B-factor (Å)	-68.675	-68.086	-49.055	-53.174	-73.160	-63.277
**Model composition**						
Chains	2		2		2	
Non-hydrogen atoms	20,520		20,923		20,319	
Protein residues	2,670		2,731		2,654	
Ligand	10		4		10	
**R.m.s. deviations**						
Bond lengths (Å)	0.032		0.012		0.012	
Bond angles (°)	2.435		2.082		2.058	
**Validation**						
MolProbity score	1.62		1.48		1.29	
Clashscore	4.63		0.79		0.64	
Favored rotamers (%)	95.88		91.23		92.65	
Poor rotamers (%)	1.37		2.30		1.83	
**Ramachandran plot**						
Favored (%)	95.98		92.32		94.41	
Allowed (%)	4.02		7.17		5.32	
Disallowed (%)	0.00		0.51		0.26	

Supplementary Table 2. Strains and Plasmids used in the study.

Strains and Plasmids	Description	Source
**Strains**		
Pseudomonas aeruginosa	strain PAO1 contains the *pchE and pchD* genes	ATCC (47085)
Escherichia coli DH10B	F^-, Δ *mcr*A (*mrr-hsd*RMS-*mcr*BC), cloning vector	Invitrogen (EC0113)
Escherichia coli BAP1	BL21(DE3) Δ*prp*RBCD::T7prom-*sfp,* T7prom-*prp*E	Reference[23]
**Plasmids**		
pET-28 a (+)	pBR322 origin, expression vector, Kan^r	Novagen (69864-3)
pPchD_nH	Derived from pET-28 a (+), with DNA region coding *pchD* gene fragment, with N-terminal 6 His Tag, Kan^r	This research (Please see the Description column and more plasmids construction details in the Methods section of the main text)
pPchE	Derived from pET-28 a (+), with DNA region coding *pchE* gene fragment, Kan^r	
pPchE_cHcSII	Derived from pPchE, with C-terminal 6 His and StrepII Tag, Kan^r	
pPchE_Y114A_cHcSII	Derived from pPchE_cHcSII, site mutant with Y114A on PchE, Kan^r	
pPchE_N368A_cHcSII	Derived from pPchE_cHcSII, site mutant with N368A on PchE, Kan^r	
pPchE_F372A_cHcSII	Derived from pPchE_cHcSII, site mutant with F372A on PchE, Kan^r	
pPchE_S472A_cHcSII	Derived from pPchE_cHcSII, site mutant with S472A on PchE, Kan^r	
pPchE_T474A_cHcSII	Derived from pPchE_cHcSII, site mutant with T474A on PchE, Kan^r	
pPchE_D480A_cHcSII	Derived from pPchE_cHcSII, site mutant with D480A on PchE, Kan^r	
pPchE_Q482A_cHcSII	Derived from pPchE_cHcSII, site mutant with Q482A on PchE, Kan^r	
pPchE_H1204A_cHcSII	Derived from pPchE_cHcSII, site mutant with H1204A on PchE, Kan^r	
pPchE_E1234A_cHcSII	Derived from pPchE_cHcSII, site mutant with E1234A on PchE, Kan^r	

Supplementary Table 3. Primers used in the study.

Primers	Sequence	Source
fPchE28_S	GATATACCATGGATCTGCCCCCCGAT	This research
fPchE28_A	GCCATATGGCTCATAGCACGCCCTCT	
V28_PchE_S	TGCTATGAGCCATATGGCTAGCATGACTG	
V28_PchE_A	GCAGATCCATGGTATATCTCCTTCT	
Tag_cHcSII_S	ACTGGAAGAGGGCGTGCTACATCATCATCACCATCACCTGCCGA<br>GCTGGAGCCATCCGCAGTTTGAAAAGTGAGCCATATGGCTAGCA	
Tag_cHcSII_A	TGCTAGCCATATGGCTCACTTTTCAAACTGCGGATGGCTCCAGCT<br>CGGCAGGTGATGGTGATGATGATGTAGCACGCCCTCTTCCAGT	
VPchE_cHS_S	TGAGCCATATGGCTAGCATGACTG	
VPchE_cHS_A	AGCACGCCCTCTTCCAGTT	
fPchD28_S	CAGCCATATGACTTCCTCGCCCGTCACCCC	
fPchD28_A	GAGGGCCGCAGGGGGTCTCATGCGCGGGCCTCCAG	
V28_PchD_S	AGACCCCCTGCGGCCCTCGAATTCGAGCTCCGTCGACAAG	
V28_PchD_A	GAGGAAGTCATATGGCTGCCGCGCGGC	
cE_Y114A_S	CTGGGTCGCGGCGCGGGCGAGGTGCTGGGCAACGT	
cE_Y114A_A	CGCGACCCAGCCACGCGGCCTGCTGCACGGAAGAC	
cE_N368A_S	CGCGGTGCCGTTGTTCGATCGGCATGC	
cE_N368A_A	CGGCACCGCGAGGAGAAATTCCGCGCTT	
cE_F372A_S	CAGACCCCGCAGGTGTGGCTCGACCACCAGCTCTA	
cE_F372A_A	GCGGGGTCTGCGCCAGCATGTCGTGGAGATCGCC	
cE_S472A_S	CAGACCCCGCAGGTGTGGCTCGACCACCAGCTCTA	
cE_S472A_A	GCGGGGTCTGCGCCAGCATGTCGTGGAGATCGCC	
cE_T472A_S	CAGGCGCCGCAGGTGTGGCTCGACCACCAGCTCTA	
cE_T472A_A	GCGGCGCCTGGCTCAGCATGTCGTGGAGATCGCC	
cE_D480A_S	CTCGCCCACCAGCTCTACCGGGTGGG	
cE_D480A_A	GGTGGGCGAGCCAGACCTGCGGGGT	
cE_Q482A_S	GCGCTGTATCGGGTGGGCGACGGTATCCT	
cE_Q482A_A	GATACAGCGCGTGGTCGAGCCAGACCTGC	
cE_H1204A_S	CGTTAGCGGCCTACGAGGCCAGCCGCGA	
cE_H1204A_A	GCCGCTAACGCGGCGAAGCTGATCACC	
cE_E1234A_S	TTACTGTGTGCGAGCCCACTGGCGCTGCTCGGTG	
cE_E1234A_A	CACACAGTAAATCAACCAGCAACAGGCGGCCCT	

Supplementary References

1. Erickson, H. P. Size and shape of protein molecules at the nanometer level determined by sedimentation, gel filtration, and electron microscopy. *Biol. Proced. Online* **11**, 32–51 (2009).
2. Sorensen, B. R. & Shea, M. A. Calcium binding decreases the stokes radius of calmodulin and mutants R74A, R90A, and R90G. *Biophys. J.* **71**, 3407–3420 (1996).
3. Katsuyama, Y. *et al.* Structural and functional analyses of the tridomain-nonribosomal peptide synthetase FmoA3 for 4-methyloxazoline ring formation. *Angew. Chem. Int. Ed.* **60**, 14554–14562 (2021).
4. Drake, E. J. *et al.* Structures of two distinct conformations of holo-non-ribosomal peptide synthetases. *Nature* **529**, 235–238 (2016).
5. Reimer, J. M. *et al.* Structures of a dimodular nonribosomal peptide synthetase reveal conformational flexibility. *Science* **366**, eaaw4388 (2019).
6. Kreitler, D. F., Gemmell, E. M., Schaffer, J. E., Wencewicz, T. A. & Gulick, A. M. The structural basis of N-acyl-α-amino-β-lactone formation catalyzed by a nonribosomal peptide synthetase. *Nat. Commun.* **10**, 3432 (2019).

7. Tanovic, A., Samel, S. A., Essen, L.-O. & Marahiel, M. A. Crystal structure of the termination module of a nonribosomal peptide synthetase. *Science* **321**, 659–663 (2008).
8. Bloudoff, K., Fage, C. D., Marahiel, M. A. & Schmeing, T. M. Structural and mutational analysis of the nonribosomal peptide synthetase heterocyclization domain provides insight into catalysis. *Proc. Natl. Acad. Sci. U. S. A.* **114**, 95–100 (2017).
9. Dowling, D. P. *et al.* Structural elements of an NRPS cyclization domain and its intermodule docking domain. *Proc. Natl. Acad. Sci. U. S. A.* **113**, 12432–12437 (2016).
10. Bloudoff, K., Rodionov, D. & Schmeing, T. M. Crystal structures of the first condensation domain of CDA synthetase suggest conformational changes during the synthetic cycle of nonribosomal peptide synthetases. *J. Mol. Biol.* **425**, 3137–3150 (2013).
11. Zhang, J. *et al.* Structural basis of nonribosomal peptide macrocyclization in fungi. *Nat. Chem. Biol.* **12**, 1001–1003 (2016).
12. Steller, S. *et al.* Initiation of surfactin biosynthesis and the role of the SrfD-thioesterase protein. *Biochemistry* **43**, 11331–11343 (2004).
13. Reimer, J. M., Aloise, M. N., Harrison, P. M. & Schmeing, T. M. Synthetic cycle of the initiation module of a formylating nonribosomal peptide synthetase. *Nature* **529**, 239–242 (2016).
14. May, J. J., Kessler, N., Marahiel, M. A. & Stubbs, M. T. Crystal structure of DhbE, an archetype for aryl acid activating domains of modular nonribosomal peptide synthetases. *Proc. Natl. Acad. Sci. U. S. A.* **99**, 12120–12125 (2002).
15. Conti, E., Stachelhaus, T., Marahiel, M. A. & Brick, P. Structural basis for the activation of phenylalanine in the non-ribosomal biosynthesis of gramicidin S. *Embo J.* **16**, 4174–4183 (1997).
16. Gulick, A. M., Starai, V. J., Horswill, A. R., Homick, K. M. & Escalante-Semerena, J. C. The 1.75 Å crystal structure of acetyl-CoA synthetase bound to adenosine-5'-propylphosphate and coenzyme A. *Biochemistry* **42**, 2866–2873 (2003).
17. Gulick, A. M., Lu, X. & Dunaway-Mariano, D. Crystal structure of 4-chlorobenzoate:CoA ligase/synthetase in the unliganded and aryl substrate-bound states. *Biochemistry* **43**, 8670–8679 (2004).
18. Hayward, S. & Berendsen, H. J. C. Systematic analysis of domain motions in proteins from conformational change: new results on citrate synthase and T4 lysozyme. *Proteins* **30**, 144–154 (1998).
19. Mori, S. *et al.* Structural basis for backbone N-methylation by an interrupted adenylation domain. *Nat. Chem. Biol.* **14**, 428–430 (2018).
20. Köksal, M., Chou, W. K. W., Cane, D. E. & Christianson, D. W. Structure of geranyl diphosphate *C*-methyltransferase from *Streptomyces coelicolor* and implications for the mechanism of isoprenoid modification. *Biochemistry* **51**, 3003–3010 (2012).
21. Wang, J. *et al.* Structural basis for the biosynthesis of lovastatin. *Nat. Commun.* **12**, 867 (2021).
22. Chen, W.-H., Li, K., Guntaka, N. S. & Bruner, S. D. Interdomain and intermodule organization in epimerization domain containing nonribosomal peptide synthetases. *ACS Chem. Biol.* **11**, 2293–2303 (2016).
23. Pfeifer, B. A., Admiraal, S. J., Gramajo, H., Cane, D. E. & Khosla, C. Biosynthesis of complex polyketides in a metabolically engineered strain of *E. coli*. *Science* **291**, 1790–1792 (2001).
