# Supplementary material for: Catalytic trajectory of a dimeric nonribosomal peptide synthetase subunit with an inserted epimerase domain
Source: Nat Commun. 2022 Feb 1;13:592. doi: 10.1038/s41467-022-28284-x (PMC8807600; doi:10.1038/s41467-022-28284-x)
Supplement: Supplementary file 3 — Reporting summary [file 41467_2022_28284_MOESM3_ESM.pdf]

nature portfolio

Corresponding author(s): Zixin Deng, Jingdan Liang, Zhijun Wang

Last updated by author(s): Dec 15, 2021

Reporting Summary

Nature Portfolio wishes to improve the reproducibility of the work that we publish. This form provides structure for consistency and transparency in reporting. For further information on Nature Portfolio policies, see our Editorial Policies and the Editorial Policy Checklist.

Statistics

For all statistical analyses, confirm that the following items are present in the figure legend, table legend, main text, or Methods section.

n/a	Confirmed	
☐	☒	The exact sample size (*n*) for each experimental group/condition, given as a discrete number and unit of measurement
☐	☒	A statement on whether measurements were taken from distinct samples or whether the same sample was measured repeatedly
☒	☐	The statistical test(s) used AND whether they are one- or two-sided<br>*Only common tests should be described solely by name; describe more complex techniques in the Methods section.*
☒	☐	A description of all covariates tested
☒	☐	A description of any assumptions or corrections, such as tests of normality and adjustment for multiple comparisons
☐	☒	A full description of the statistical parameters including central tendency (e.g. means) or other basic estimates (e.g. regression coefficient) AND variation (e.g. standard deviation) or associated estimates of uncertainty (e.g. confidence intervals)
☒	☐	For null hypothesis testing, the test statistic (e.g. *F*, *t*, *r*) with confidence intervals, effect sizes, degrees of freedom and *P* value noted<br>*Give P values as exact values whenever suitable.*
☒	☐	For Bayesian analysis, information on the choice of priors and Markov chain Monte Carlo settings
☒	☐	For hierarchical and complex designs, identification of the appropriate level for tests and full reporting of outcomes
☒	☐	Estimates of effect sizes (e.g. Cohen's *d*, Pearson's *r*), indicating how they were calculated

Our web collection on statistics for biologists contains articles on many of the points above.

Software and code

Policy information about availability of computer code

Data collection	CryoEM data collection used SerialEM-3.8 software in Titan Krios equipped with a K2 Direct camera.
Data analysis	HHpred server (based on HMM-HMM comparison, https://toolkit.tuebingen.mpg.de/tools/hhpred, version: 57c8707149031cc9f8edceba362c71a3762bdbf8), RELION 3.0, MotionCor2_1.2.6, Gctf_v1.18, Chimera-1.13.1, ChimeraX-1.1.1, Pymol-2.2.3, Rosetta_2018.48.60516_bundle, RosettaDock in Rosetta v3.2, coot-0.8.9.2, Phenix-1.18.1-3865-000, Molprobity-4.5.1, Agilent OpenLAB CDS ChemStation Edition-C.01.06, Agilent Masshunter Acquisition-B.06.00, Agilent Masshunter Qualitative Analysis Navigator-B.08.00, HOLLOW-1.3, cryoDRGN-0.3.0, DynDom-1.5, and ISOLDE-1.1.0.

For manuscripts utilizing custom algorithms or software that are central to the research but not yet described in published literature, software must be made available to editors and reviewers. We strongly encourage code deposition in a community repository (e.g. GitHub). See the Nature Portfolio guidelines for submitting code & software for further information.

Data

Policy information about availability of data

All manuscripts must include a data availability statement. This statement should provide the following information, where applicable:

- Accession codes, unique identifiers, or web links for publicly available datasets
- A description of any restrictions on data availability
- For clinical datasets or third party data, please ensure that the statement adheres to our policy

All of the 3D cryo-EM density maps in this study have been deposited in the Electron Microscopy Data Bank with accession numbers EMD-31198, EMD-31201, EMD-31199, EMD-31202, EMD-31200, and EMD-31203. The atomic coordinates have been deposited in the Protein Data Bank as 7EMY, 7EN1, and 7EN2.

Field-specific reporting

Please select the one below that is the best fit for your research. If you are not sure, read the appropriate sections before making your selection.

- [x] Life sciences
- [ ] Behavioural & social sciences
- [ ] Ecological, evolutionary & environmental sciences

For a reference copy of the document with all sections, see nature.com/documents/nr-reporting-summary-flat.pdf

Life sciences study design

All studies must disclose on these points even when the disclosure is negative.

Sample size	The sample sizes were determined by counting the total number of movie stacks and protein particles. No predetermined sample size calculation was performed. These sample sizes were chosen by collecting all the useful movie stacks and protein particles which contain high-resolution features, and they were sufficient because the resolution of the cryo-EM maps resulting from them is high enough for building the atomic models. A total of 3749 movie stacks were collected. After Rounds of 2D and 3D classification for particle selection and refinements, a total of 393,498 particles were further processed, leading to the 3.67 anstrom PchE consensus density map. More detailed processes were depicted in Methods and Supplementary Figs. 3-5.
Data exclusions	The "bad" particles which did not produce good quality 2D class averages or 3D reconstructions were excluded. This is a standard image process which is widely accepted in the cryoEM community.
Replication	Reproducibility relies on the large number of particles used for 2D averages and 3D reconstructions. The quality and resolution is measured by gold standard Fourier shell correlation curves. All gel images shown are representative of replicates. At least three independent gel electrophoresis experiments were performed, and all attempts at replication were successful.
Randomization	The raw particles were randomly selected by program RELION 3.0. (based on the template)
Blinding	The investigators were blinded during data collection and analysis.

Reporting for specific materials, systems and methods

We require information from authors about some types of materials, experimental systems and methods used in many studies. Here, indicate whether each material, system or method listed is relevant to your study. If you are not sure if a list item applies to your research, read the appropriate section before selecting a response.

Materials & experimental systems

n/a	Involved in the study
☒	☐ Antibodies
☒	☐ Eukaryotic cell lines
☒	☐ Palaeontology and archaeology
☒	☐ Animals and other organisms
☒	☐ Human research participants
☒	☐ Clinical data
☒	☐ Dual use research of concern

Methods

n/a	Involved in the study
☒	☐ ChIP-seq
☒	☐ Flow cytometry
☒	☐ MRI-based neuroimaging
