# Supplementary material for: Catalytic trajectory of a dimeric nonribosomal peptide synthetase subunit with an inserted epimerase domain
Source: Nat Commun. 2022 Feb 1;13:592. doi: 10.1038/s41467-022-28284-x (PMC8807600; doi:10.1038/s41467-022-28284-x)
Supplement: Supplementary file 4 — Description of Additional Supplementary Files [file 41467_2022_28284_MOESM4_ESM.pdf]

Description of Additional Supplementary Files

File Name: Supplementary Movie 1

Description: A morph movie demonstrating the Cy substrate tunnel switch (ArCP entry side) controlled by the key residue F372.

File Name: Supplementary Movie 2

Description: The trajectory of PchE (highlighted with one chain) describing the changes between the catalytic steps by a series of 120 cryo-EM maps reconstructed by cryoDRGN software (left, PCA projection; middle, cryo-EM maps; right, schematics).
